# Knockout of MMP3 Weakens Solid Tumor Organoids and Cancer Extracellular Vesicles

**DOI:** 10.3390/cancers12051260

**Published:** 2020-05-16

**Authors:** Eman A. Taha, Chiharu Sogawa, Yuka Okusha, Hotaka Kawai, May Wathone Oo, Abdellatif Elseoudi, Yanyin Lu, Hitoshi Nagatsuka, Satoshi Kubota, Ayano Satoh, Kuniaki Okamoto, Takanori Eguchi

**Affiliations:** 1 Department of Dental Pharmacology, Okayama University Graduate School of Medicine, Dentistry and Pharmaceutical Sciences, 2-5-1 Shikata-cho, Okayama 700-8525, Japan; pj7l8pfb@s.okayama-u.ac.jp (E.A.T.); caoki@md.okayama-u.ac.jp (C.S.); yokusha@bidmc.harvard.edu (Y.O.); riku21@s.okayama-u.ac.jp (Y.L.); k-oka@okayama-u.ac.jp (K.O.); 2 Department of Medical Bioengineering, Okayama University Graduate School of Natural Science and Technology, Okayama 700-8530, Japan; Ayano113@cc.okayama-u.ac.jp; 3 Department of Biochemistry, Ain Shams University Faculty of Science, Cairo 11566, Egypt; 4 Division of Molecular and Cellular Biology, Department of Radiation Oncology, Beth Israel Deaconess Medical Center, Harvard Medical School, Boston, MA 02115, USA; 5 Department of Oral Pathology and Medicine, Okayama University Graduate School of Medicine, Dentistry and Pharmaceutical Sciences, Okayama 700-8525, Japan; de18018@s.okayama-u.ac.jp (H.K.); p1qq7mbu@s.okayama-u.ac.jp (M.W.O.); jin@okayama-u.ac.jp (H.N.); 6 Department of Biochemistry and Molecular Dentistry, Okayama University Graduate School of Medicine, Dentistry and Pharmaceutical Sciences, Okayama 700-8525, Japan; abdellatif.abouelseoud@umontreal.ca (A.E.); kubota1@md.okayama-u.ac.jp (S.K.); 7 Centre Hospitalier Universitaire Sainte-Justine Hospital Research Center, University of Montreal, Québec, QC H3T 1C5, Canada; 8 Department of Dental Anesthesiology, Okayama University Graduate School of Medicine, Dentistry and Pharmaceutical Sciences, Okayama 700-8525, Japan; 9 Advanced Research Center for Oral and Craniofacial Sciences, Okayama University Graduate School of Medicine, Dentistry and Pharmaceutical Sciences, Okayama 700-8525, Japan

**Keywords:** matrix metalloproteinase 3 (MMP3), extracellular vesicles (EVs), tumoroid, tumor organoid, tumorigenesis, three-dimensional (3D) culture system

## Abstract

The tumor organoid (tumoroid) model in three-dimensional (3D) culture systems has been developed to reflect more closely the in vivo tumors than 2D-cultured tumor cells. Notably, extracellular vesicles (EVs) are efficiently collectible from the culture supernatant of gel-free tumoroids. Matrix metalloproteinase (MMP) 3 is a multi-functional factor playing crucial roles in tumor progression. However, roles of MMP3 within tumor growth and EVs have not unveiled. Here, we investigated the protumorigenic roles of MMP3 on integrities of tumoroids and EVs. We generated MMP3-knockout (KO) cells using the CRISPR/Cas9 system from rapidly metastatic LuM1 tumor cells. Moreover, we established fluorescent cell lines with palmitoylation signal-fused fluorescent proteins (tdTomato and enhanced GFP). Then we confirmed the exchange of EVs between cellular populations and tumoroids. LuM1-tumoroids released large EVs (200–1000 nm) and small EVs (50–200 nm) while the knockout of MMP3 resulted in the additional release of broken EVs from tumoroids. The loss of MMP3 led to a significant reduction in tumoroid size and the development of the necrotic area within tumoroids. MMP3 and CD9 (a category-1 EV marker tetraspanin protein) were significantly down-regulated in MMP3-KO cells and their EV fraction. Moreover, CD63, another member of the tetraspanin family, was significantly reduced only in the EVs fractions of the MMP3-KO cells compared to their counterpart. These weakened phenotypes of MMP3-KO were markedly rescued by the addition of MMP3-rich EVs or conditioned medium (CM) collected from LuM1-tumoroids, which caused a dramatic rise in the expression of MMP3, CD9, and Ki-67 (a marker of proliferating cells) in the MMP3-null/CD9-low tumoroids. Notably, MMP3 enriched in tumoroids-derived EVs and CM deeply penetrated recipient MMP3-KO tumoroids, resulting in a remarkable enlargement of solid tumoroids, while MMP3-null EVs did not. These data demonstrate that EVs can mediate molecular transfer of MMP3, resulting in increasing the proliferation and tumorigenesis, indicating crucial roles of MMP3 in tumor progression.

## 1. Introduction

Extracellular vesicles (EVs) are lipid bi-layered vesicles released from cells under physiological and pathological conditions. EVs are key players in intercellular communications at local and distant sites [1]. EVs carry various molecular cargos such as nucleic acids, proteins, lipids, and metabolites [1,2,3,4]. EVs are often classified into: (1) exosomes which are secreted by exocytosis of intra-luminal vesicles via membrane fusion of multivesicular bodies with plasma membrane and their size range between 50 to 200 nm, (2) ectosomes aka microvesicles or microparticles formed by cytoplasmic membrane budding, and fission; their size typically ranges from 200 nm to 1000 nm in diameter [5,6,7]. These populations of EVs are often distinguished based on their biogenesis, size, content, and function [1,8,9]. In addition to these EV types, other vesicles have been reported including; oncosomes, large oncosomes (1–10 μm) [10,11], matrix vesicles [12,13,14], migrasomes (50 nm to 3 μm) [15,16], exopheres (~4 μm), exomeres (~35 nm), and bacterial outer membrane vesicles (OMV) [4,17,18,19]. Since EVs are heterogeneous populations, there is no unanimous consensus on the nomenclature of them. General terms such as “exosomes” and “microvesicles” have been broadly used. The International Society of Extracellular Vesicles (ISEV) proposed to use the term EVs in general to describe vesicles naturally released from the cells and surrounded by a lipid bilayer [20]. In the present study, we will use the term EVs in general and classify them into small EVs (s-EVs) sized 50–200 nm and large EVs (L-EVs) sized 200–1000 nm. Notably, recent studies, including ours, have shown that tumor-derived EVs can reprogram the recipient cells by transferring their bioactive cargo into them [21,22,23]. In the present study, we aimed to explore the roles of tumor-derived EVs in tumor development in vitro.

The matrix metalloproteinase (MMP) family consists of 28 members that share similarities in their structure, regulation, and function [24]. The prototypical MMPs consists of three principal domains; (1) A pro-domain that functions as an intramolecular inhibitor to maintain the enzyme in an inactive state, (2) a catalytic domain that promotes the proteolytic activity, and (3) a hemopexin-like repeat domain (PEX), which determines the substrate specificity [25,26,27,28,29]. Earlier studies showed that MMPs constituted a large family of zinc/calcium-dependent endopeptidases, playing key roles in extracellular matrix (ECM) remodeling because of their ability to degrade numerous components of ECM (such as collagens and proteoglycans) and non-ECM proteins (such as adhesion molecules, cytokines, protease inhibitors, and membrane receptors) [24]. Later studies have shown that matrix metalloproteinase 3 (MMP3) was a bifunctional protein that acted as a proteolytic enzyme and a transcriptional factor, playing crucial roles in tumor progression [30,31,32]. Therefore, we defined MMP3 as a moonlighting protein, as it plays multiple roles in the cells as a proteinase as well as a transcription factor [32,33]. It has been shown that some members of MMPs were expressed at high levels in particular cancer types and promoted tumor progression. We have compared protumorigenic phenotypes of rapidly metastatic murine cancer cell line LuM1 with a parental cancer cell line Colon26 (aka CT26) [31,32,34,35,36] and shown that MMP3 and MMP9 were highly expressed in LuM1 cells. The RNA interference (RNAi)-mediated knockdown of MMP3 and/or MMP9 significantly attenuated tumor growth and metastasis in the tumor allograft mouse model [31]. Most recently, we established MMP3-knockout (KO) cells using CRISPR/Cas9 genome editing technology on the LuM1 cells and then compared the oncogenic effects of MMP3-KO cells versus LuM1 cells [32]. This study indicated that knockout of *Mmp3* results in a significant inhibition of tumor growth in vivo, cellular migration and invasion in vitro [32]. However, a mechanism of how MMP3 enriched EVs influences the characteristics of EVs and tumors has not been completed yet. We investigate this issue in the present study.

The two-dimensional (2D) cell culture system has been frequently used for cancer research and drug screening [37]. In conventional 2D culture systems, cells are cultured as monolayers on flat surfaces of plates, which allow each cell to access the same amount of growth factors and nutrients present in the medium, resulting in homogenous growth and proliferation [38]. Besides, the strong physical interaction present between cells and 2D culture substrates resulted in alteration in the tumor cell behaviors that differ from those of tumors growing in vivo [37]. Thus, the 2D culture model fails to correctly mimic the proper tissue architecture and complex microenvironment in vivo [39]. To overcome the limitations of the 2D culture system, the three-dimensional (3D) cell culture model (aka a spheroid or organoid culture) has been developed to closely mimic in vivo tissue microenvironments [39,40]. The 3D culture model maintains the interactions between cells and their ECM, create gradient access of oxygen and nutrient, and buildup a combination of tissue-specific scaffolding cells [41]. Similar to human cancers, proliferating, quiescent, and dying cells coexist in normoxic, hypoxic, or necrotic zones within tumor organoids [34,42,43]. Thus, the 3D tumor models reflect more closely the in vivo human tumors, which prompted us to define tumor organoids as “tumoroids.” Among several methodologies of tumoroid models, we have adopted gel-free tumoroid models cultured on NanoCulture Plates (NCP) and ultra-low attachment (ULA) plates [34,35,36,42,44]. A great advantage of the gel-free tumoroid model is the collectability of the secretome including EVs from their culture supernatants. NCP is a nanopatterned gel-free scaffold for 3D cell culture [45]. The mogul field structure on NCPs restricts cells to sprawl on the base and enable tumor cells to migrate from a scaffold to another scaffold more actively than cells cultured on the 2D plate. The increased migration and lesser attachment of cancer cells on the NCPs enable tumor cells forming 3D tumoroids [34,35,36,42,44]. ULA plates have been also useful for the collection of secretome including EVs. Cells do not rapidly migrate on ULA plates compared to NCPs. We have examined a few types of culture media such as serum-containing media versus serum-free stemness-enhancing media in combination with the 3D culture systems. In vitro culture of tumoroids in such a 3D nano-environment combined with a defined stem cell medium enabled the cells to grow slowly and form large organoids that expressed multiple stem cell markers and intercellular adhesion molecules [34,42]. However, EVs derived from the 3D tumoroids model have not well characterized yet. Moreover, the roles of tumoroid-derived EVs in tumor development have not unveiled. We, therefore, investigate these issues in the present study.

Several fluorescence-based methods have been developed to monitor EV biogenesis, transmission (or uptake), distribution, and subcellular localization. Such materials and methods for EV analysis include lipid-based fluorescence labeling [34,46], transmembrane proteins (i.e., CD63) fused with fluorescent proteins [8,47], and a membrane lipid-binding palmitoylation (palm) signal fused with fluorescent proteins such as tandem dimer Tomato (tdTomato) or enhanced green fluorescence protein (EGFP) [48]. In the present study, we utilize the palm system and abbreviate as palmGFP (palmG) and palmtdTomato (palmT). Protein S-acylation is a lipid modification that enables the covalent attachment of long-chain palmitic fatty acids to thiol groups of cysteine residues through a thioester linkage [49,50]. This type of protein modification is commonly known as S-palmitoylation that allows the association of proteins with cellular membranes [51]. Therefore, the fusion of fluorescent proteins with the palmitoylation sequence enables the whole-cell membrane labeling [52,53]. As EVs are generated from cellular membrane components [4], we assumed that tagging the cellular membrane component with palm-fluorescent proteins would enable the labeling of EVs.

Here, we explore (i) the protumorigenic role of MMP3 on the in vitro tumoroid formation under the 3D culture system and on their EVs integrity. We also explore (ii) whether MMP3-rich or MMP3-null EVs could alter the tumoroid formation and examine (iii) the EV-mediated molecular transfer of MMP3 into the MMP3-KO tumoroids under the 3D culture system.

## 2. Results

### 2.1. MMP3 Knockout Reduces the Release of CD9 and CD63 within Extracellular Vesicles

**Figure 1:**
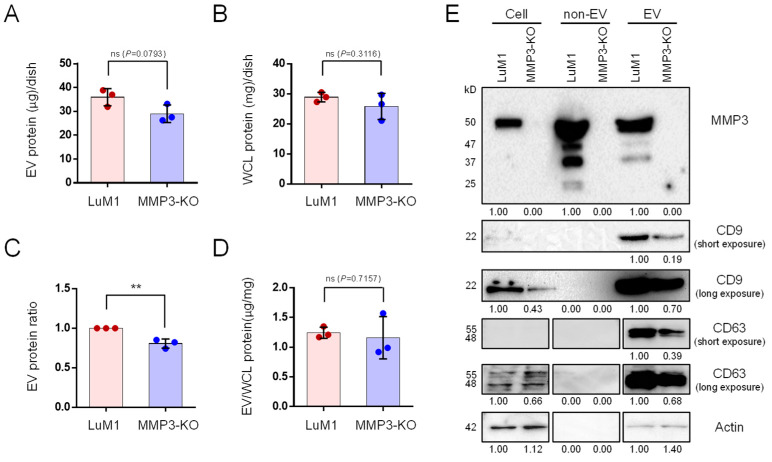
MMP3 knockout reduces the release of CD9 and CD63 within extracellular vesicles. Tumoroids were formed in 10-cm ultra-low attachment (ULA) plates for 6 days. Extracellular vesicle (EV) and non-EV fractions were collected from the culture supernatants. (**A**,**B**) The total protein concentration in the (**A**) EV and (**B**) whole cell lysate (WCL) fractions of LuM1-tumoroids and MMP3-KO tumoroids. (**C**) Relative EV protein ratio comparing two cell lines. (**D**) EV protein concentration per the WCL proteins. ** *p* < 0.01; ns, not significant. (**E**) Western blotting showing MMP3, CD9, CD63, and β-actin in tumoroids, non-EV, and EV fractions. The 54-kD bands indicate the full-length MMP3, the 47-kD bands represent the active form consists of the catalytic, hinge, and PEX domains, the 37-kD represents the catalytic domain, and the 25-kD shows the PEX domain of MMP3. The expression level of β-actin was examined as a loading control as well as to check if it was released from cells. The protein amounts loaded from WCL and EV fractions were 10 µg per lane, while 5 µg per lane were loaded from the non-EV fractions. The experiments were repeated twice. For full images of Western blotting, see Figure S1.

To explore the role of MMP3 on the cellular communication in cancer, we generated an MMP3-knockout cell line from a murine high metastatic cancer cell line LuM1 using the CRISPR/Cas9 genome editing system [32]. The release of EV proteins from MMP3-KO-tumoroids tended to decrease compared to LuM1-tumoroids (Figure 1A–D). However, there was no statistically significant difference in the protein concentration of the whole cell lysate (WCL) between LuM1 and MMP3-KO-tumoroids.

MMP3 was markedly detected in the cellular, non-EV, and EV fractions of the LuM1, while the complete loss of MMP3 was confirmed in MMP3-knockout LuM1 cells, non-EV (including soluble proteins) and EV fractions (Figure 1E, top row; Figure S1A), suggesting a successful knockout of the *Mmp3* gene. Next, we examined CD9 (a category-1 EV marker protein) expression pattern. Interestingly, the CD9 content was significantly down-regulated in both cellular and EV fractions of the MMP3-null cells compared to their counterpart (Figure 1E, second and third rows; Figure S1B). Moreover, CD63 (also a category-1 EV marker) was reduced in the EV fraction of the MMP3-null cells (Figure 1E, fourth and fifth rows; Figure S1C). Recently, we have shown that GAPDH and β-actin were released in the EV and non-EV soluble fractions upon membrane-damaging cell stress [21,54]. Therefore, we examined the expression levels of β-actin and GAPDH not only as a loading control but also to investigate whether they were released within EVs or non-EV soluble proteins. Our results revealed that both β-actin and GAPDH were detected in the EVs derived from both cell lines, but not in the non-EV fractions (Figure 1E, bottom lane; Figure S1D,E; Figure S2A). Notably, β-actin levels were considered as a loading control for WCL and EV fractions, whereas GAPDH was not (Figure S1D,E). Furthermore, the SDS-polyacrylamide gel was stained with Coomassie brilliant blue (CBB) after the electrophoretic separation (Figure S2B).

These data demonstrate that MMP3 controls the secretion of CD9/CD63-contained EVs.

### 2.2. MMP3 Knockout Impacts Physical Integrities of Extracellular Vesicles

**Figure 2:**
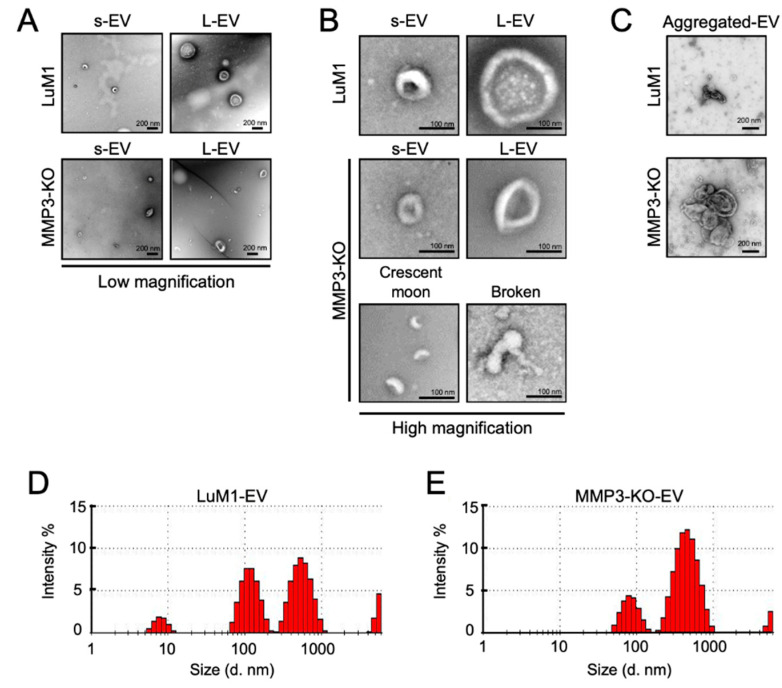
MMP3 knockout impacts the physical integrities of extracellular vesicles. (**A**–**C**) TEM images of EV fractions derived from the LuM1 and MMP3-KO tumoroids. at (**A**) low magnification, (**B**) high magnification, and of (**C**) aggregated EVs. s-EVs, small EVs; L-EVs, large EVs. Scale bars; 200 nm (in low magnification) and 100 nm (in high magnification). (**D**,**E**) Representative histograms showing the particle diameter distributions of EVs derived from (**D**) LuM1 tumoroids and (**E**) MMP3-KO tumoroids. The experiments were repeated twice.

Further, we examined the morphology and size of EVs secreted from “tumoroids” by transmission electron microscopy (TEM) and Zetasizer, respectively. Both LuM1- and MMP3-KO tumoroids released two types of EVs—small EVs (s-EVs) ranged approximately 50–200 nm and large EVs (L-EVs) more broadly ranged between 200 and 1000 nm (Figure 2A,B, Table 1). According to their size, the s-EVs were supposed to be exosomes, while the L-EVs were supposed to be microvesicles.

Meanwhile, crescent moon-like shaped and broken EVs were particularly seen in the MMP3-KO EV fraction released by MMP3-KO tumoroids (Figure 2A,B). Additionally, we observed large aggregates (500–800 nm) of EVs derived from MMP3-KO compared to their counterparts (Figure 2C).

Particle diameter distribution analysis using Zetasizer revealed that the size of both s-EVs (peaked at 84 nm) and L-EVs (peaked at 465 nm) released from MMP3-KO tumoroids were smaller than those (peaked at 119 nm and 561 nm, respectively) of LuM1 tumoroids (Figure 2D,E). The small particles that were detected as 5–10 nm diameters in the LuM1-EV fraction might be damaged membrane vesicles or lipoprotein particles e.g., HDL (5–12 nm) which have similar size ranges as EVs [55].

These findings demonstrate that MMP3 is important for maintaining the physical integrities of EVs, and the loss of MMP3 resulted in disorganizing the EVs structures.

### 2.3. Loss of the Mmp3 Gene Reduces the In Vitro Tumorigenicity

**Figure 3:**
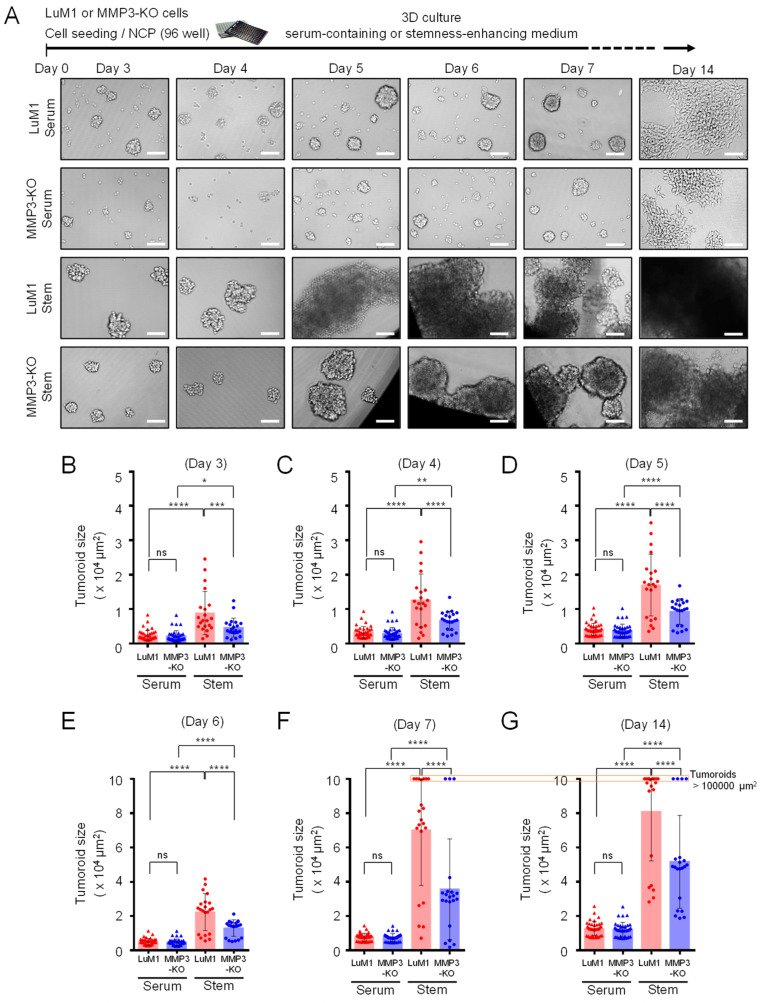
Loss of the *Mmp3* gene reduces the in vitro tumorigenicity. Tumoroids were formed in the NanoCulture Plate (NCP)-based 3D culture with a serum-containing or stemness-enhancing medium. (**A**) Representative images of the LuM1 and MMP3-KO tumoroids. The upper panel shows the experimental design. (**B**–**G**) Tumoroid size quantification on (**B**) day 3, (**C**) day 4, (**D**) day 5, (**E**) day 6, (**F**) day 7, and (**G**) day 14 of the tumoroid growth periods. * *p* < 0.05, ** *p* < 0.01, *** *p* < 0.001, **** *p* < 0.0001; ns, not significant; *n* = 39 (LuM1 serum, MMP3-KO serum), *n* = 21 (LuM1 stem, MMP3-KO stem). The alternative graphs of Figure 3F,G with tumoroid plotting larger than 100,000 μm^2^ are shown in Figure S3. The experiments were repeated twice.

Next, we examined the consequences of *Mmp3* loss on the tumoroid formation. The LuM1 and MMP3-KO cells were cultured in the 3D culture system either under serum-containing or stemness-enhancing conditions for 14 days. Larger tumoroids were formed in the stemness-enhancing medium compared to smaller tumoroids seen in the serum-containing medium (Figure 3A–G).

A highly significant difference in the tumoroid size between LuM1 and MMP3-null cells was observed under the stemness-enhancing culture conditions from day 3 until day 14 (Figure 3A–G; Figure S3). The size of MMP3-null tumoroids was significantly smaller compared to LuM1 tumoroids (Figure 3B,G; Figure S3).

**Figure 4:**
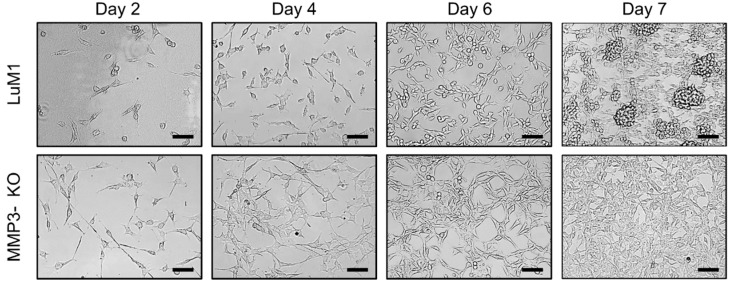
Representative images of re-cultured LuM1 and MMP3-KO cells in 2D culture. Tumoroids on day 14 were trypsinized and re-cultured under the 2D culture condition in serum-containing media. Scale bars, 100 µm. The experiment was repeated twice.

Subsequently, we asked whether MMP3-KO and LuM1 cells were able to grow if they were reseeded under the 2D culture conditions or not. We trypsinized tumoroids and reseeded under the 2D culture condition. Both cell types proliferated and reached confluency by day 7. More interestingly, LuM1 cells were able to grow into tumoroids even under the 2D culture condition, whereas MMP3-null cells were not (Figure 4).

These data suggest that the stemness-enhancing medium promotes the tumorigenic aggregation of tumor cells, whereas the serum-containing medium stimulates the cellular differentiation and decreasing the fusion of tumoroids. Besides, these findings indicate that loss of MMP3 has a great significance on inhibiting the tumoroid formation in vitro.

### 2.4. The Addition of MMP3-Rich EVs Accelerated the In Vitro Tumorigenesis of MMP3-KO Cells

We further investigated whether treating the MMP3-KO tumoroids with LuM1-EVs (MMP3-rich) or MMP3-KO-EVs (MMP3-null) would foster the in vitro tumorigenesis under the 3D culture system. A protocol has been shown that seeding the cells first followed by the addition of EVs two days later and centrifuging the plate improves the uptake of EVs into cells [48]. As a pilot study, we examined this protocol to see the effect of three different concentrations (1.25, 2.5, and 5 μg/mL) of LuM1-EVs on the growth or cytotoxicity in MMP3-KO tumoroids. With this protocol, we found that the addition of 5 μg/mL of EVs significantly promoted the tumoroid growth compared to the lower concentrations of EVs (Figure S4).

**Figure 5:**
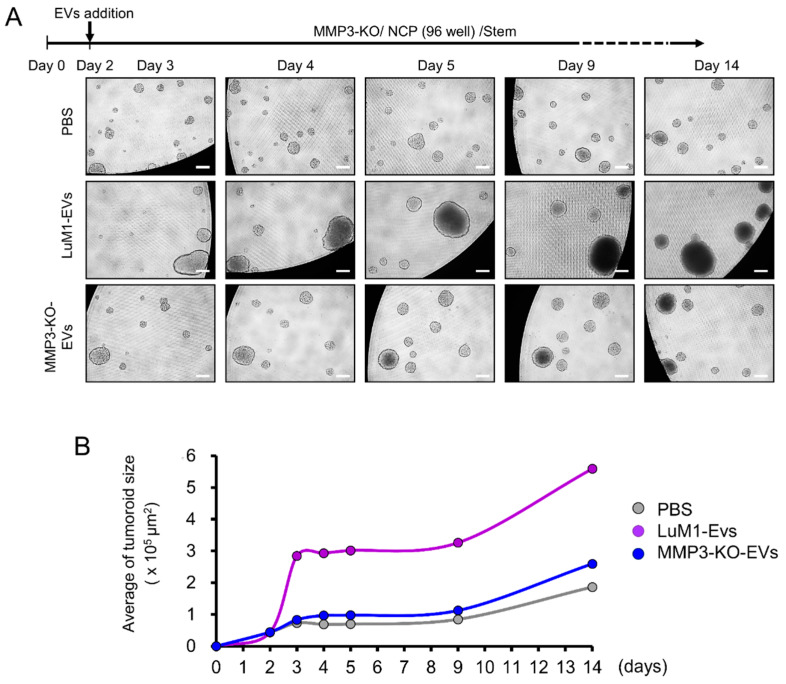
The addition of MMP3-rich EVs accelerated the in vitro tumorigenesis of MMP3-KO cells. MMP3-KO tumoroids were treated with PBS, LuM1-EVs, or MMP3-KO-EVs at a final concentration of 5 μg/mL in the NCP-based 3D culture with the stemness-enhancing medium. (**A**) Experimental scheme (top) and representative photomicrographs (bottom) of tumoroid maturation at the indicated timepoints. Scale bar, 100 µm. (**B**) A time plot graph showing the average size of the MMP3-KO tumoroids following the different treatments over the indicated time points. See the next figure for statistical analysis.

We then found that the addition of LuM1-EVs tended to increase the size of tumoroids from the next day of the EVs addition to 12 days later, while the addition of MMP3-KO-EVs might not promote the tumoroid growth (Figure 5A). The growth of MMP3-KO tumoroids following the different treatments was monitored by plotting the average tumoroid size over the time following the different treatments (Figure 5B). The addition of LuM1-derived EVs fostered the tumoroids growth compared to the other two groups.

**Figure 6:**
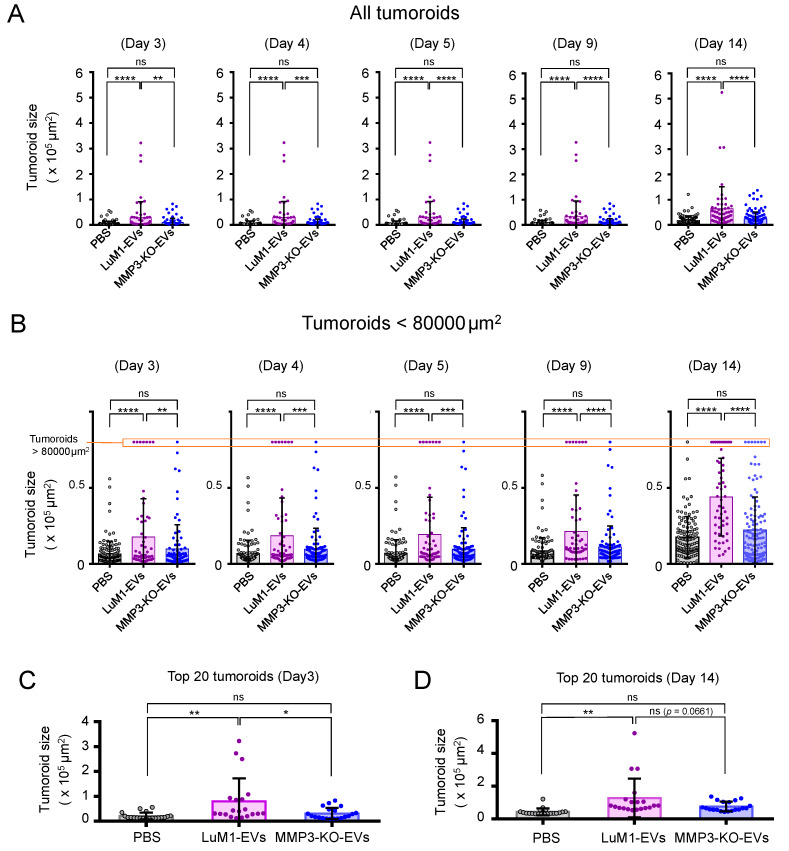
Tumoroid size altered by the addition of LuM1-EVs versus MMP3-KO-EVs. (**A**,**B**) Tumoroid size quantification of (**A**) all tumoroids and (**B**) tumoroids smaller than 80,000 µm^2^ at the indicated time points of the tumoroid formation. Cell aggregates (>500 µm^2^) were considered as tumoroids. (**B**) Tumoroids larger than 80,000 µm^2^ were shown on the top of the graph. (**C**,**D**) Top 20 tumoroids size quantification on (**C**) day 3 and (**D**) day 14. * *p* < 0.05, ** *p* < 0.01, *** *p* < 0.001, **** *p* < 0.0001; ns, not significant; *n* = 54–130 (number of plots). The experiments were repeated twice.

Notably, the addition of LuM1-EVs resulted in significant increases in the size of tumoroids from the next day of the EVs addition to 12 days later, while the addition of MMP3-KO-EVs did not have any impact on the tumoroid growth (Figure 6A,B). In parallel, the comparison of the top 20 tumoroid sizes in the three groups revealed that the addition of LuM1-derived EVs significantly potentiated the formation of enlarged tumoroids compared to the other treatments with MMP3-KO-EVs or PBS (Figure 6C,D).

We thus found that that (i) MMP3-high, LuM1-derived EVs augmented the tumor growth *in vitro* and (ii) the loss of MMP3 in EVs diminished the protumorigenic properties of the EVs.

### 2.5. Establishment of Fluorescent-Labeled LuM1 and MMP3-Null Cells

**Figure 7:**
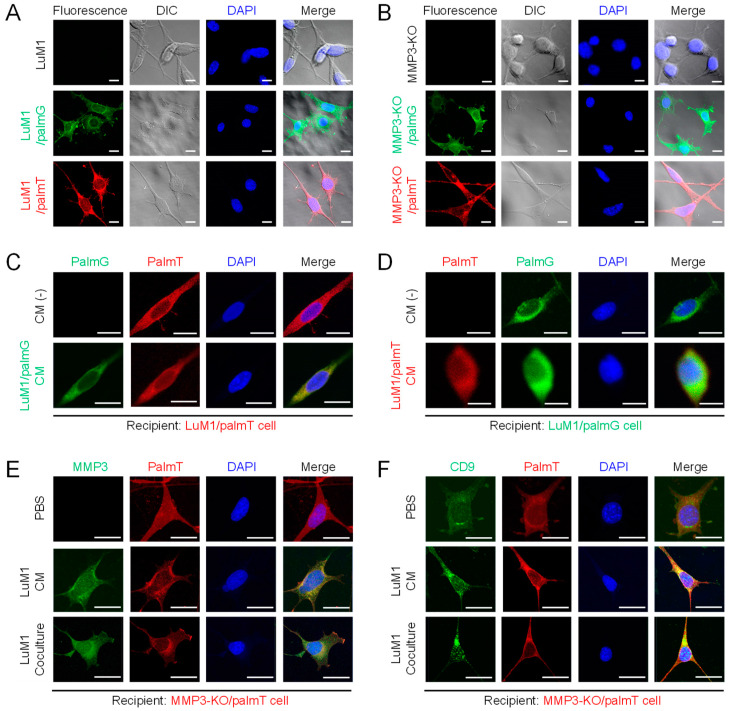
Establishment of fluorescent-labeled LuM1 and MMP3-KO cells. (**A**,**B**) Fluorescent labeled (**A**) LuM1 and (**B**) MMP3-KO cells. The stable cells were established by the transfection of expression constructs for palmT (red) and palmG (green) fluorescent proteins tagged with the palmitoylation signal. Images were taken using confocal laser scanning microscopy (CLSM). Non-fluorescent LuM1 and MMP3-KO cells were used as negative controls. (**C**,**D**) Molecular transfer of (**C**) palmG- and (**D**) palmT-labeled EVs from the conditioned medium (CM) of donor cells to recipient cells. (**C**) LuM1/palmT cells were treated with/without the CM of LuM1/palmG cells. (**D**) LuM1/palmG cells were treated with/without the CM of LuM1/palmT cells. (**E**,**F**) Immunostaining of (**E**) MMP3 and (**F**) CD9 in recipient MMP3-KO/palmT cells stimulated with PBS, LuM1-CM, or coculturing with LuM1 cells in the Transwell system. DNA was stained with DAPI (blue). DIC, differential interference contrast. Scale bars, 20 µm. The experiments were repeated twice.

It has been shown that EVs were derived from the plasma membrane [4] and could be labeled with palmitoylation signal-tagged fluorescent proteins [48]. To monitor the tumor EVs-uptake and exchange between cell populations, the plasma membrane of the LuM1 and MMP3-KO cells were labeled with palmitoylation signal-fused fluorescent reporters, namely palmG and palmT, thereby green and red labeled cells were established (Figure 7A,B).

Next, we confirmed the exchange of EVs between the cells. Two different colored fluorescent cells LuM1/palmG (green) and LuM1/palmT (red) cells were treated with each other conditioned media (CM) collected under the 2D culture condition. In this EVs exchange assay, if the red/green recipient cells have taken up the green/red-EVs from donor cells, an increase in the green/red fluorescence signals should be observed, whereas, in non-treated cells, no green/red fluorescence signals should be detected. Indeed, the green/red fluorescence was markedly detected in the cells treated with the CM of green/red cells, indicating that EVs were exchanged between the cells (Figure 7C,D).

Additionally, we confirmed the EVs-mediated molecular transfer of MMP3 and CD9 under the 2D culture system by treating MMP3-KO cells with LuM1-CM or by co-culturing with the MMP3 produced by the LuM1 cells in the transwell insert. Interestingly, MMP3 was restored and detected in the cytoplasmic and nuclear regions of MMP3-KO recipient cells after the addition of LuM1-CM or coculturing (Figure 7E).

The CD9 expression level was low in the MMP3-KO cells as shown in Figure 1. However, CD9 was significantly increased in the nuclear and cytoplasmic regions of MMP3-KO recipient cells after the addition of LuM1-CM or coculturing (Figure 7F). *Mmp3* was deleted at the genome level, while *Cd9* was not in the MMP3-KO cells. Therefore, as data interpretation, there were two possibilities including (i) CD9 was transferred from LuM1-EVs to recipient cells and/or (ii) endogenous CD9 was induced in the recipient cells after the stimulation with LuM1-EVs.

Collectively, these data prove the successful labeling and exchanging of EVs between cell populations.

### 2.6. Penetration of MMP3-Rich EVs into Organoids

The bidirectional EVs-mediated transfer of cargo effectively influences the recipient phenotype to promote the development of an environment hospitable toward the cancer growth, invasion, and metastasis [56]. Moreover, the roles of EVs in the intercellular communication within the tumor microenvironment is increasingly acknowledged. Therefore, we examined whether MMP3 enriched EVs and CM was transferred and penetrating MMP3-null recipient tumoroids by immunohistochemistry. MMP3 was well detected on the surface and inside of the recipient tumoroids after the addition of LuM1-EVs and LuM1-CM (data not shown) [57]. Additionally, MMP3-null tumoroids contained more space between cells and thus more fragile, while the addition of MMP3-rich, LuM1-EV, or –CM promoted the formation of solid tumoroids. To examine the molecular transfer and penetration of MMP3 into tumoroids, we next stained the recipient tumoroids by immunofluorescence (IF). To eliminate non-specific reaction, we confirmed the specificity of the anti-MMP3 antibody in the LuM1/palmT tumoroids (Figure S5).

**Figure 8:**
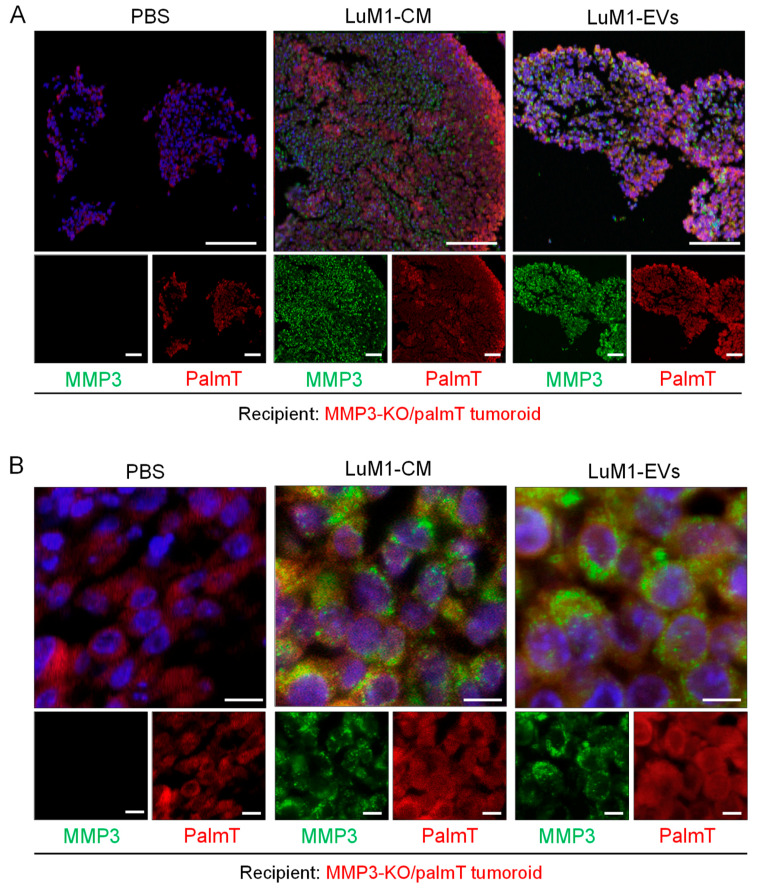
The EV-mediated deep transfer of MMP3 into tumoroids. MMP3-KO/palmT (red) tumoroids were treated with PBS, LuM1-CM, or LuM1-EVs for 24 h in the ULA-based 3D culture system. MMP3 (green) was stained by immunofluorescence. Nuclei were stained blue with DAPI. Images were taken by CLSM. (**A**) low and (**B**) high magnifications were shown. Top images, merge of three color. Scale bars; 100 µm (in low magnification images) and 10 µm (in high magnification images).

To examine molecular penetration and tissue localization of MMP3, we used the CLSM. MMP3 from LuM1-EVs and –CM was found to penetrate the MMP3-null tumoroids (Figure 8A). Notably, intracellular and intranuclear penetration of MMP3 in the recipient tumoroids was seen after the addition of MMP3-rich, LuM1-EVs (Figure 8A,B). MMP3 transferred from EVs and CM were seen as speckles in cytoplasm and nuclei in the recipient MMP3-null tumoroids (Figure 8B).

**Figure 9:**
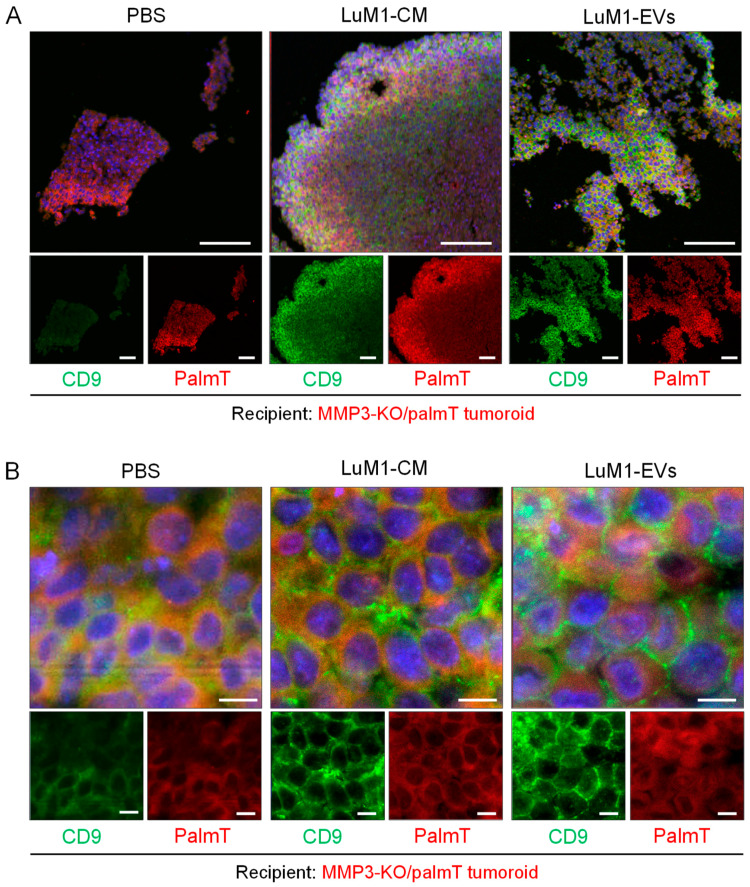
Treatment with LuM1-derived EVs and CM recovered CD9 in MMP3-null tumoroids. MMP3-KO/palmT (red) tumoroids were treated with PBS, LuM1-CM, or LuM1-EVs for 24 h in the ULA-based 3D culture system. CD9 (green) was stained by immunofluorescence. Nuclei were stained blue with DAPI. Images were taken by CLSM. (**A**) low and (**B**) high magnifications were shown. Top images, merge of three color. Scale bars; 100 µm (in low magnification images) and 10 µm (in high magnification images).

We have found that CD9 was decreased in MMP3-KO cells as shown in Figure 1 and Figure 7. Next, we examined whether CD9 could be altered in the CD9-low, MMP3-KO recipient tumoroids by adding LuM1-EVs or -CM. Indeed, CD9 was well stained in the recipient tumoroids, especially the parts close to the surface of tumoroids after the addition of the MMP3-rich CM or EVs (Figure 9A). Moreover, CD9 and endogenous palmT in the recipient tumoroids became abundantly expressed on the cell surface and well co-localized seen as honeycomb shape, suggesting that CD9 contributed to cell-cell adhesion in the recipient tumoroids (Figure 9B).

These findings indicate that LuM1-derived EVs and CM enhanced the solidity of MMP3-null tumoroids, which were relatively fragile. Additionally, MMP3 carried by LuM1-EVs was highly penetrative and deeply transferred to the recipient MMP3-null tumoroids. The intranuclear transfer of MMP3 and the increase in CD9 could contribute to the increased solidity in the MMP3-KO tumoroids.

### 2.7. The Knockout of the Mmp3 Gene Significantly Decreased the Transmissive Potential of Tumoroid-Derived EVs

**Figure 10:**
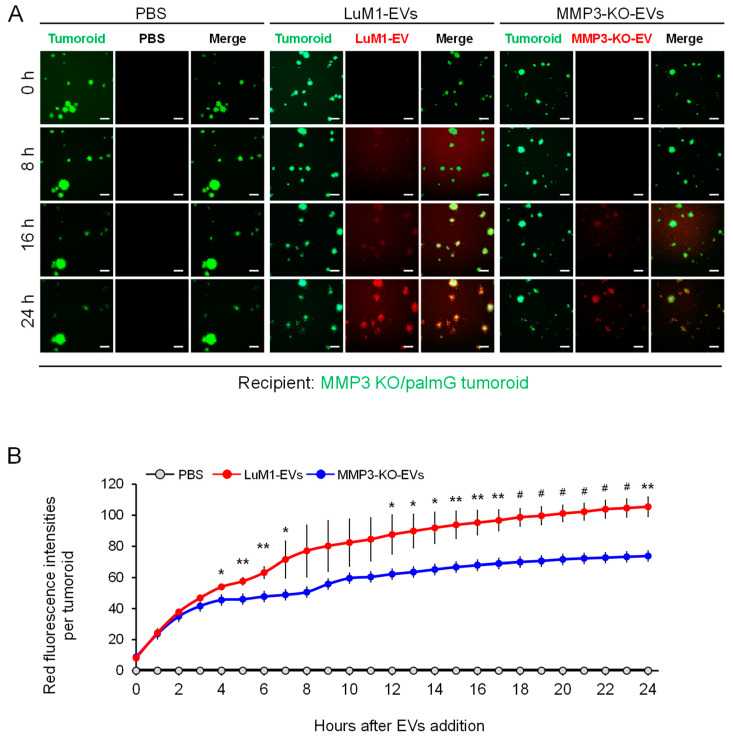
The Knockout of the MMP3 significantly decreased the transmissive potential of tumoroid-derived EVs. EVs were collected after 6 days from the culture supernatants of tumoroids that formed in ULA plates. EVs were fluorescently labeled with BODIPY TR Ceramide (red). The labeled EVs or PBS were added to the MMP3-KO/palmG (green) tumoroids at a concentration of 5 μg/mL in the NCP-based 3D culture with the stemness-enhancing medium. The uptake of EVs was monitored over 24 h using the high contents screening (HCS) system. (**A**) Time-course imaging of EV uptake (red) by MMP3-KO/palmG tumoroids (green) for 24 h. Scale bar, 100 µm. (**B**) Red fluorescence intensities of transmitted EVs in MMP3-KO/palmG tumoroids. The average fluorescence intensity of the PBS treatment group at time point 0 h was evaluated as background and subtracted from raw values. *n* = 3, * *p* < 0.05, ** *p* < 0.01, and # *p* < 0.001 (LuM1-EVs versus MMP3-KO-EVs).

We have developed a method to examine the EV transfer to tumoroids by labeling EVs with red-fluorescent sphingolipids [34]. In the present study, we monitored whether LuM1 tumoroid-derived or MMP3-KO tumoroid-derived heterogenous EVs (shown in Figure 1) were differently transferred to the MMP3-null tumoroids over 24 h. We found that the MMP3-null tumoroids rapidly internalized the MMP3-rich, LuM1-EVs at a highly significant rate compared to the MMP3-null EV from 4 h to 24 h post-EVs addition (Figure 10A,B). Furthermore, the EVs uptake by tumoroids was increased in a time-dependent manner for 24 h (Figure 10B).

To sum up, these findings indicate that MMP3-rich, LuM1 tumoroid-derived EVs were highly transmissive and associated with tumoroids, while the loss of MMP3 in tumoroid-EVs reduced the transmissive and binding properties. These data also support our hypothesis that both endogenous and exogenous MMP3 play key roles in promoting the tumorigenesis, thereby MMP3-rich EVs were rapidly taken up by the MMP3-null tumoroids.

### 2.8. MMP3-Rich EVs and CM Rescue the Cell Proliferation of MMP3-KO Tumoroids

**Figure 11:**
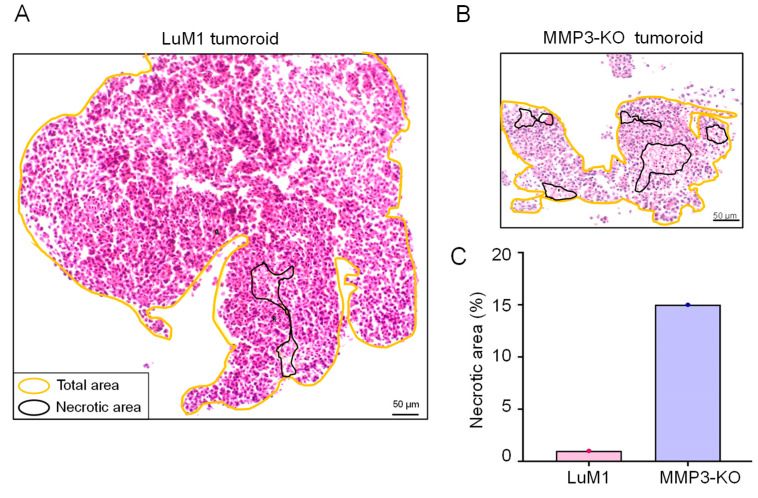
MMP3-knockout resulted in necrotic cell death in tumoroids. Tumoroids were cultured in the ULA-based 3D culture system with a stemness-enhancing medium for 8 days. (**A**,**B**) Hematoxylin and eosin (H&E) staining of (**A**) LuM1- and (**B**) MMP3-KO tumoroids. Necrotic areas were enclosed with black color, while the total tumoroids area was enclosed with yellow color. Scale bars, 50 µm. (**C**) The percentage of the necrotic areas in both tumoroids.

In the course of the present study, we compared the morphological characteristics of the LuM1 tumoroids versus MMP3-KO tumoroids by histological (H&E) staining. Five necrotic areas were observed in the MMP3-KO tumoroid, whereas only one small necrotic area was found in the LuM1 tumoroid (Figure 11A,B). The development of necrotic areas in the MMP3-KO tumoroid was at a higher percentage compared to the LuM1 tumoroid (15% versus 1% of the total area, respectively) (Figure 11A–C). The summed total and the percentage of necrotic areas were larger in the MMP3-KO tumoroids compared to their counterpart (Table 2).

**Figure 12:**
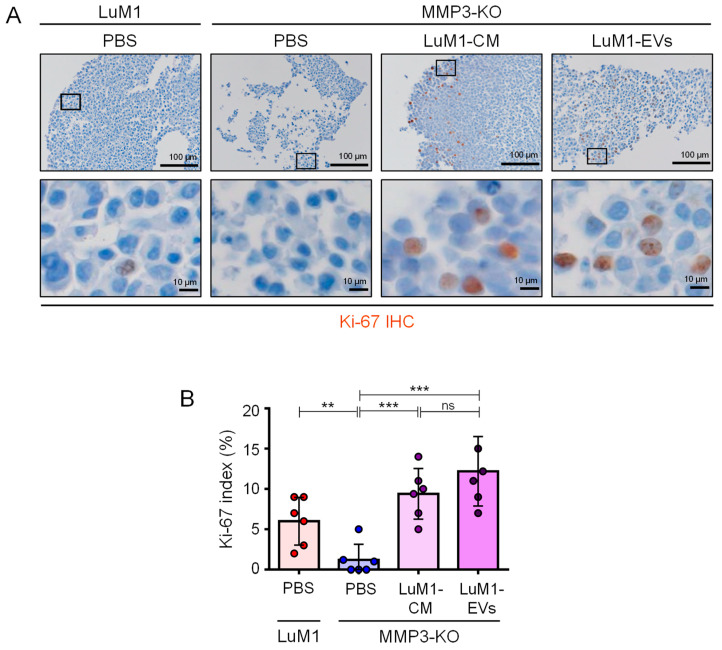
MMP3 enriched-EVs and CM rescue the proliferation of MMP3-KO tumoroids. LuM1-tumoroids or MMP3-KO tumoroids were treated with PBS, LuM1-CM, or LuM1-EVs for 24 h, and then Ki-67 was immunostained. (**A**) Ki-67 immunostaining (brown) in the LuM1 and MMP3-KO tumoroids. Scale bars; 100 µm (in low magnification images) and 10 µm (in high magnification images). (**B**) Ki-67 labeling index (%). *n* = 6, ** *p* < 0.01, *** *p* < 0.001; ns, not significant. Experiments were repeated twice.

Additionally, there was a significant reduction in the Ki-67 expression, a marker of cell proliferation, in the MMP3-KO cells compared to their counterparts (Figure 12A,B). Following the addition of MMP3-rich CM and EVs, the recipient MMP3-KO tumoroid displayed a highly proliferative phenotype as judged by the highly significant increase in Ki-67 expression index (Figure 12A,B). These findings prove that MMP3 plays a crucial role in promoting cell proliferation in tumoroids and delaying the necrotic process.

## 3. Discussion

### 3.1. Summary

**Figure 13:**
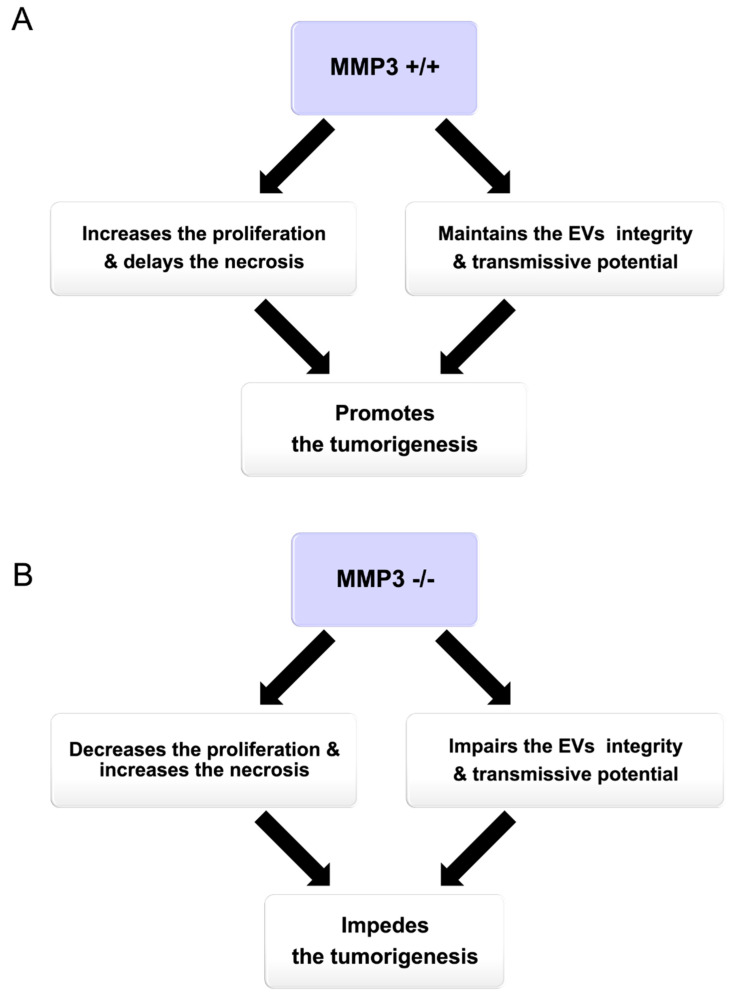
Graphical abstract summarizing the role of MMP3 on tumorigenesis in vitro. (**A**) Illustrating the net results of the presence and (**B**) absence of MMP3 protein.

MMP3 is a proteolytic enzyme, as well as a transcriptional factor that plays a crucial role in tumor progression [30,31,32]. However, the roles of MMP3 within EVs had not unveiled before our study. We recently generated MMP3-KO cells by CRISPR/Cas9 system [32] and analyzed their properties in EVs and tumorigenesis. In the current study, we found that MMP3 was abundantly detected in the high-metastatic cancer cells, their non-EV fluids, and EVs, but not in/from MMP3-KO cells. Thus, we newly explored (i) the oncogenic role of MMP3 on the in vitro tumoroid formation and on their EVs integrity under the 3D culture system, (ii) the tumorigenic potential of MMP3-rich versus MMP3-null EVs, and (iii) the EVs-mediated molecular transfer of MMP3 into the MMP3-KO tumoroids under the 3D culture system (Figure 13).

### 3.2. Potential Mechanism of How MMP3 Promotes Tumorigenesis

Our study indicates that MMP3 contained in EVs promotes primary tumorigenesis and metastasis also called secondary tumorigenesis. Several studies have reported that several MMP family members were packaged in EVs from body fluids or various types of cell lines [31,58,59,60,61,62]. Our current results are consistent with recent data that LuM1-EVs (defined as oncosomes) enriched with MMP3 were highly transmissive and protumorigenic in vitro and in vivo [32]. MMP3 is one of epithelial-to-mesenchymal transition (EMT) markers in cancer metastasis [63], and it is well-known that MMP3 makes cancer cells detached from solid mass and transferred to distant regions of body [31]. In the current study, we focused on the roles of MMP3 in EVs of cancer cell lines for evaluating its tumorigenic potential. As a potential mechanism of the tumorigenesis, MMP3 in EVs can penetrate to recipient cells resulting in inducing transformation (normal to cancer cells). We have shown that MMP3 could penetrate cell nuclei and transactivate connective tissue growth factor gene [CTGF aka cell communication network factor 2 (CCN2)] by interacting with DNA and heterochromatin proteins (HP1/CBXs) [30,32]. Moreover, we recently showed that MMP3 contained within EVs penetrate recipient cells and their nuclei [32]. MMP3-rich EVs were able to transactivate CCN2 gene promoter, while knockout of MMP3 from the EVs abolished this transactivating effect. The induction of CCN2 could be a key mechanism by which MMP3 induces transformation and tumor progression, as the stromal expression of CTGF promotes angiogenesis and prostate cancer tumorigenesis [64]. Moreover, CCN2 modulates cell cycle progression through the upregulation of cyclin A [65]. Besides, CTGF is associated with oncogenic activities in glioblastoma by inducing the expression of the antiapoptotic proteins, Bcl-xl, survivin, and Flip [66]. Thus, the protumorigenic effect of MMP3 could be partially mediated by the induction of this multi-functional factor, CTGF.

Intranuclear MMP3 can also trans-activate HSP genes encoding cytoprotective factors, in collaboration with HP1/CBXs [67]. Thus, intranuclearly translocated MMP3 could regulate broader intranuclear proteins and genes, some of which could be involved in cellular transformation such as EMT. Our current data indicated that MMP3 might also regulate CD9 and CD63 at transcriptional or post-transcriptional levels. The transactivating role of MMP3-EVs on the CCN2 gene was strictly mediated by a cis-element called TRENDIC [32]. Therefore, it would be important to verify which gene promoters contain TRENDIC-like motifs directly bound by MMP3. Some target genes regulated by MMP3 may involve cellular transformation.

Moreover, intranuclearly penetrating MMP3 could initiate cellular transformation by cleaving particular intranuclear proteins. It has become clear that the function of MMPs was not only restricted to degrade or inactivate matrix proteins and that proteolysis by MMPs can modulate or even increase functions of substrate proteins [68]. Simultaneously, EV-associated MMP3 could activate extracellular transforming signals such as TGFβ by cleaving their pro-forms or inhibitory factors. Activities of many proteins are positively regulated by MMP proteolysis including CCN2/CTGF [32,69,70], insulin growth factor binding proteins (IGFBPs) heparin-binding epidermal growth factor (HB-EGF) [71], fibroblast growth factor receptor 1 (FGFR1) [72], interleukin-1beta (IL-1β) [73], and tumor necrosis factor-alpha (TNF-α) [74]. Such proteins activated by MMPs strengthen our in vitro findings that MMP3 can foster tumor development by modulating the activities of many signaling pathways and their receptors. We are currently investigating such mechanisms of transformation induced by MMP3.

### 3.3. Potential Role of MMP3 in Cytoprotection

Our data suggest that MMP3 contained in EVs plays a cytoprotective role in tumors. Knockout of MMP3 markedly increased necrotic area in tumors thereby inhibiting tumor growth. The addition of MMP3-rich EVs rescued tumor growth by increasing proliferating cells. Therefore, it is conceivable that MMP3 has cytoprotective effects and stimulates cell proliferation in tumors. Such cytoprotective and proliferative roles of MMP3 could be mediated by downstream factors such as HSPs and CTGF/CCN2 [30,67]. We showed that intranuclear MMP3 can trans-activate HSP genes encoding cytoprotective factors, in collaboration with HP1/CBXs [67]. Besides, CCN2 modulates cell cycle progression through the upregulation of cyclin A [65].

Indeed, the MMP3-null tumoroid size was significantly smaller than their parental counterpart. Besides, the necrotic onset was occurring at a higher rate in the MMP3-null tumoroid compared to the LuM1 tumoroids. Necrosis is accidental death of cells that is induced in response to extreme physiological conditions, such as hypoxia, toxin exposure, ischemia, reactive oxygen species exposure, nutrient deprivation, and extreme temperature changes [75,76]. Indeed, inside tumoroids/tumors are hypoxic and deprived of nutrients [34,42,77], although some cytoprotective factors could protect cells against necrotic cell death. Morphologically, necrotic cell death is characterized by swelling of the cellular organelles, a process of oncosis (also called ischemic cell death), and early plasma membrane rupture leading to loss of intracellular content [78]. The cell death observed in tumoroids in the current study fits the morphological criteria of necrosis.

Our data also indicated that MMP3 is a regulator for the physical and biological characteristics of EVs. Tetraspanins CD9 and CD63, category-1 EV markers, were downregulated in MMP3-null EVs compared to their counterparts, suggesting that MMP3-knockout reduced the endogenous production or stability and subsequent release of CD9/CD63-contained EVs. It has been known that tetraspanins, CD326/EpCAM (these are category-1 EV markers), and the tight junction protein claudin-7 partners associate with each other for cell–cell adhesion and apoptosis resistance [79]. Moreover, tetraspanin interaction with another tetraspanin and integrins often depended on palmitoylation [80,81]. Therefore, MMP3-KO-triggered loss of CD9 and CD63 could reduce the cell-cell and EV-cell adhesions required for tumoroid integrity.

### 3.4. Release of L-EVs and s-EVs from 3D Tumoroids

The morphological visualization of EVs showed abnormal disorganized shapes of EVs such as, crescent moon-like and broken EVs that were associated with the *Mmp3* loss. These data indicated that MMP3 is necessary for maintaining the stability of structural proteins required for the integrity of EVs. Besides, we distinguished two subpopulations of 3D tumoroid-derived EVs, namely s-EVs (50–200 nm) and L-EVs (200–1000 nm). It is worth noting that, under the 2D culture system both LuM1 and MMP3-KO cells secreted homogeneous intact s-EVs (50–300 nm diameter) [32]. This inconsistency is because our current study was performed under the 3D culture system which is completely different from the 2D culture system. Thus, intra-tumoral hypoxia developed under the 3D culture model may stimulate the production of L-EVs. The release of both s-EVs and L-EVs might be a signature characteristic of the 3D tumoroids, a model resembling of tumors in vivo. It has been reported that adipocytes secreted L-EVs containing cytoskeletal proteins and molecular chaperones, whereas s-EVs were shown to contain ECM proteins [82]. Moreover, the proteomic analysis of s- and L-EVs derived from a colorectal cancer cell line revealed that s-EVs were enriched with proteins associated with cell-matrix adhesion and cell–cell junctions [83]. Similarly, s-EVs from fibrosarcoma cells showed similar enrichment for adhesion proteins [83]. Likewise, one of our recent studies has demonstrated that the prostate cancer (PC-3) cells release two types of vesicles, s-EVs (30–200 nm) under a non-heated condition, L-EVs (200–500 nm) and membrane-damaged EVs which were associated with HSP90α expression [21]. Notably, both membrane-damaged EVs and L-EVs were co-released upon the heat shock stress, suggesting that vesicular membranes were damaged by the stress [21]. Thus, the two different EVs populations may play distinct biological roles in the recipient cells. We are currently challenging to separate the s- and L-EVs from the tumoroids and distinguish their properties.

### 3.5. Fluorescent Labeling of EVs

To monitor the transmission and uptake of EVs between cells, we used two systems (i) PalmGFP and PalmtdTomato (Figure 7) or (ii) BODIPY TR ceramide labeled EVs (Figure 10). By utilizing the first system, we detected a robust fluorescent signal of donor-derived EVs in recipient cells, indicating the bidirectional exchange of EVs between the cells. Of note, PalmGFP and PalmtdTomato EV labeling strategy were designed to visualize and track multiple EV subtypes. Furthermore, by using these reporters, 0.22- and 0.8-μm sized EV populations, as well as sucrose density gradient with EV-marker proteins (such as Alix) were observed [48]. Through the second EVs monitoring system, we tracked the uptake rate of MMP3-rich versus MMP3-null EVs for 24 h. An increase in BODIPY TR ceramide/EVs signal was noticed as early as 3 h post-EVs exposure period, and the increase continued until reached the saturation level after 24 h (Figure 10). Thus, both fluorescent-EV monitoring systems were useful for monitoring the EV exchange. It has been shown that uptake of EVs occurred via multiple routes, such as the direct fusion between EVs and the plasma membrane [84], as well as EV internalization through lipid raft-, clathrin-, and caveolae-dependent endocytosis, macropinocytosis, and phagocytosis [85,86,87,88,89,90]. However, it is unclear which mechanism(s) is employed in different cell types under various conditions. 

### 3.6. Inter MMPs Regulation

Besides, EV-derived MMPs could promote proteolysis in recipient cells leading to tumor progression. Wang et al. indicated that EVs derived from adipocytes promoted lung cancer metastasis via transferring MMP3 that resulted in increasing the MMP9 activity in lung cancer cells [91]. It has been shown that one MMP can activate another MMP including other members of MMPs. Therefore, the high expression of active MMP3 and/or MMP9 could activate other MMPs. Indeed, we have shown that both MMP3 and MMP9 were expressed at high levels in LuM1 cells and proved their important roles in tumor progression [31]. Nevertheless, the loss of MMP3 was crucial to reduce tumor and EV development. Our data indicate that exogenous MMP3 can be positioned at a higher level in the protease cascade that promotes tumor progression in the recipient cells.

## 4. Materials and Methods

### 4.1. Cells

A rapidly metastatic murine cancer cell line LuM1 [34,36,92] and MMP3-KO cells line [32] were maintained in RPMI-1640 with 10% fetal bovine serum (FBS) and penicillin, streptomycin, and amphotericin B. MMP3-KO cells were established using the CRISPR/Cas9 system from the LuM1 cell line [32]. Briefly, Cas9 protein and guide RNA that targets *Mmp3* exon 1 were co-transfected into LuM1 and stable MMP3-KO clones with frame-shifting deletion were obtained.

### 4.2. Tumoroid Culture

Tumoroids were formed in the 3D culture systems using NanoCulture Plate (NCP) (Medical & Biological Laboratories, Nagoya, Japan) or ultra-low attachment (ULA) culture plates/dishes (Greiner, Kremsmunster, Austria) within mTeSR1 stem-cell medium (Stemcell Technologies, Vancouver, BC, Canada) or the above-mentioned serum-containing medium as described previously [34,35,36,42].

For quantification of tumoroids size and number, cells were seeded in a 96 well NCP for 14 days at a concentration of 5.0 × 10^3^ cells in 200 μL mTeSR1 or RPMI-1640 media with 10% FBS. Tumoroid maturation was monitored every day and photographed using the Floid cell imaging station (Thermo Fisher, Waltham, MA, USA) from day 1 until day 7 and a BZ-X microscope (Keyence, Osaka, Japan) starting from day 10 until the end of the experiment day 14. The tumoroid size was measured using Image J software (NIH, Bethesda, MD, USA).

### 4.3. Re-Seeding Assay

Tumoroids were cultured in the 3D and stem-cell medium condition for 14 days and detached by trypsin/EDTA. The detached cells were re-seeded in a 24-well 2D culture plate at a concentration of 1.5 × 10^4^ cells/well in RPMI-1640 with 10% FBS. The cell images were taken by using the Floid cell imaging station (Thermo Fisher, Waltham, MA, USA) on days 2, 4, 6, and 7 after the seeding.

### 4.4. Preparation of EVs and Conditioned Media

Tumoroid-derived EVs were used for tumoroid formation assays. Otherwise, 2D cultured cells-derived CM was used for 2D experiments. EVs were prepared from culture supernatants of tumoroids using a modified polymer-based precipitation method [21,22,93]. Briefly, cells were seeded on a 10-cm ULA dish at a density of 1.0 × 10^6^ cells/8 mL mTeSR1 medium and cultured for 6 days. The formed tumoroids were washed with PBS (-), and then further cultured in serum-free medium (4 mL per dish) for 2 days. Cell culture supernatant was collected and centrifuged at 2000 × *g* for 30 min at 4 °C to remove detached cells. The supernatant was then centrifuged at 10,000 × *g* for 30 min at 4 °C to remove cell debris. The supernatant (8 mL) was concentrated to less than 1 mL by using an Amicon Ultra-15 Centrifugal Filter Devices for M.W. 100k (Merck Millipore, Burlington, MA, USA). The concentrate was applied to the Total EVs Isolation System (Thermo Fisher, Waltham, MA, USA). The pass-through was concentrated using an ultrafiltration device for molecular weight 10 kD and used as a non-EV fraction. The EV fraction was suspended in 100 µL PBS (-) and used 3D-tumoroid-EVs. Protein concentration was measured using a micro BCA protein assay kit (Thermo Fisher, Waltham, MA, USA).

For immunofluorescence in the 2D culture system, culture supernatants were collected from serum-free media of 2D-cultured donor cells during the exponential growth phase (70% confluence). The culture supernatants were centrifuged at 2000× *g* for 15 min to get rid of cells and debris, followed by diluting in a ratio 1:1 with a fresh culture medium. The CM was stored at −80 °C. Recipient cells were treated with the CM for 48 h.

### 4.5. Transmission Electron Microscopy

As described previously [21,42], a 400-mesh copper grid coated with formvar/carbon films was hydrophilically treated. The EVs suspension (5–10 µL) was placed on Parafilm, and the grid was visualized at 5000, 10,000, or 20,000 times magnification with an H-7650 transmission electron microscope (TEM) (Hitachi, Tokyo, Japan) at the Central Research Laboratory, Okayama University Medical School.

### 4.6. Particle Diameter Distribution

As described previously [21,22], 40 µL of EV fraction within PBS (-) was used. Particle diameters of the EV fractions in a range between 0 and 6000 nano-diameters were analyzed in Zetasizer nano ZSP (Malvern Panalytical, Malvern, UK).

### 4.7. Western Blotting

Western blotting was performed as described [21,93]. Cells were cultured for 6 days on a 6-well ULA plate at a density of 3.0 × 10^5^ cells/3 mL mTeSR1 medium in a well. Cells were further cultured in serum-free media for 2 days. On day 8, the supernatants and tumoroids were collected, centrifuged at 2000× *g*, 4 °C for 5 min. The supernatants were used for EV preparations as mentioned above. To prepare WCL, tumoroids were lysed in a RIPA buffer (1% NP-40, 0.1% SDS, and 0.5% deoxycholate, and EDTA-free protease inhibitor cocktail in PBS) using 25-gauge syringes. The cell lysate was incubated for 30 min on ice and then centrifuged at 12,000× *g* for 20 min at 4 °C. The preparation method of EV and non-EV fraction was described above. The supernatant was used as WCL. The same protein amounts for each lane were subjected to sodium dodecyl sulfate-polyacrylamide gel electrophoresis (SDS-PAGE), followed by transfer to a polyvinylidene fluoride (PVDF) membrane using a semi-dry transfer system. The membranes were blocked in 5% skim milk in Tris-buffered saline containing 0.05% Tween 20 for 60 min at room temperature (RT) and then incubated overnight with rabbit monoclonal antibodies; anti-MMP3 (EP1186Y, ab52915, Abcam, Cambridge, UK), anti-CD9 (EPR2949, ab92726, Abcam, Cambridge, UK) or anti-CD63 (EXOAB-CD63A-1, System Biosciences). For CD63, blocking was performed in 10% overnight and the primary antibody was reacted for 2 days. The membranes were incubated with horseradish peroxidase (HRP)-conjugated secondary antibodies. For GAPDH, the HRP-conjugated anti-GAPDH mouse monoclonal antibody (HRP-60004, Proteintech, Rosemont, IL, USA) was used. For Actin, anti-Actin rabbit antibody (A2066, Sigma Aldrich, St. Louis, MO, USA) was utilized. Blots were visualized with the ECL substrate (Merck Millipore, Burlington, MA, USA).

We loaded 10 µg protein per lane from the WCL and EV, while 5 µg from non-EV fractions were used. To obtain relative expression levels, relative band intensities were measured in the same membrane using Image J. The raw values were converted into relative values as control = 1.0. For normalization of the WCL, relative values of the protein of interest per loading control were calculated. For EV and non-EV, the raw values of the protein of interest in the MMP3-KO cells were divided by its counterpart relative values of LuM1 cells.

### 4.8. Coomassie Brilliant Blue Staining

Protein samples (1 µg each) were loaded on the SDS-PAGE. After the electrophoresis run, the gel was stained with Coomassie Brilliant Blue R-250 solution (1610436, Bio-Rad, Hercules, CA, USA) for 30 min with gentle agitation followed by washing with the destaining solution (50% methanol, 10% glacial acetic acid) for 2 h until the background became less dark.

### 4.9. EV-Driven In Vitro Tumorigenesis

MMP3-KO cells were seeded at 5.0 × 10^3^ cells/200 μL mTeSR1 medium in a well of 96-well NCP. After two days, EVs derived from 3D-tumoroids (LuM1 or MMP3-KO) were added to MMP3-null tumoroids at a final concentration of 5 μg/mL. Then the plate was centrifuged at 1800× *g* for 1 h at 4 °C to increase the internalization of EVs into the tumoroids [48].

The MMP3-KO tumoroids maturation was monitored over 14 days using a microscope FSX100 (Olympus Life Science, Tokyo, Japan). Then tumoroid size was measured using Image J.

### 4.10. Palm Fluorescent Cells

The lentiviral reporter constructs of CSCW-palmitoylation signal-tandem dimer Tomato (palmT) and CSCW-palmitoylation signal-EGFP (palmG) were kindly gifted from Dr. Charles P. Lai [48]. For virus production, HEK293T cells at 70–80% confluence were transfected with PalmT or PalmG constructs, psPAX2 packaging plasmid, and pMD2.G envelope plasmid using PEI max (Polysciences). LuM1 or MMP3-KO cells were infected by using the spinfection method with the viral solution. Infected/transduced stable cells were selected using puromycin. Isolation of single clones was carried out by limiting dilution method. We established palmtdTomato-expressed LuM1 cells (designated LuM1/palmT), palmGFP-expressed LuM1 cells (designated LuM1/palmG), palmGFP-expressed MMP3-KO cells (designated MMP3-KO/palmT), and palmGFP-expressed MMP3-KO cells (designated MMP3-KO/palmG).

To confirm fluorescent labeling, the palm fluorescent cells were seeded on a type I collagen-coated coverslip in a 24-well plate at a density of 1 × 10^4^ cells/well in a serum-containing culture media and cultured for 48 h.

### 4.11. EVs Exchange Assay

Two different colored fluorescent cells (LuM1/palmG and LuM1/palmT cells) were used as donor cells or recipient cells with each other in the 2D culture system. The donor cells were seeded at 1 × 10^6^ cells in a 6 cm dish and cultured overnight in a serum-containing culture media. The grown cells of 70–80% confluence were washed twice with PBS, and then the culture media was replaced with serum-free medium and cultured for a further 2 days. The culture supernatant was collected and centrifuged at 2000× *g* for 15 min at 4 °C to remove detached cells and the supernatants were diluted in a ratio 1:1 with a fresh culture medium and used as CM. Recipient cells were seeded on a type I collagen-coated coverslip inserted in a 24-well plate at a density of 1 × 10^4^ cells/well and cultured for 24 h in a serum-containing culture media. The recipient cells were treated by donor cells-derived CM for 48 h.

For coculturing, recipient MMP3-KO cells were seeded on coverslips. LuM1-donor cells (1 × 10^4^ cells/well) were seeded on a culture insert with a 0.45-μm pore (Greiner, Kremsmunster, Austria) in a 24-well plate. The insert with donor cells was placed on the well containing the recipient cells and cocultured for 48 h.

### 4.12. Confocal Laser-Scanning Microscopy

Cells were fixed in 4% paraformaldehyde (PFA) for 10 min at RT and permeabilized with 0.5% Tween-20 for 10 min. For blocking the non-specific reaction of primary antibodies, cells were blocked in 10% normal goat serum solution (Dako, Tokyo, Japan) for 30 min, then incubated overnight at 4°C with rabbit anti-MMP3 antibody (EP1186Y, ab52915, Abcam, Cambridge, UK) or rabbit anti-CD9 antibody (EPR2949, ab92726, Abcam, Cambridge, UK), for overnight at 4 °C and subsequently with a secondary antibody, goat anti-rabbit IgG Alexa Fluor 488 (A-11034, Thermo Fisher, Waltham, MA, USA) for 1 h at RT. As a negative control, the same protocol was performed without primary antibody staining. Washes after antibody reactions were done with PBS, three times for 3 min each, on a shaker at RT. The mounting and DNA staining was performed by using Immunoselect Antifading Mounting Medium DAPI (SCR-038448, Dianova, Germany). Fluorescent images were taken using a confocal laser scanning microscopy LSM780 (Carl Zeiss, Oberkochen, Germany) at Central Research Laboratory, Okayama University Medical School.

### 4.13. Immunofluorescence of Tumoroids

For tumoroid formation, cells were cultured for 6 days at a density of 3.0 × 10^5^/3 mL mTeSR1 in a well of a 6-well ULA plate. Then tumoroids were treated with PBS, the 3D-tumoroid LuM1-EVs at a final concentration of 5 μg/mL or the 3D-tumoroid LuM1-CM (diluted 1:1 with fresh mTeSR1) for 24 h. Then the plate was centrifuged at 1800× *g* for 1 h at 4 °C to increase the internalization of EVs into the tumoroids [48]. Tumoroids were washed with PBS and fixed in 4% PFA for 10 min. Tumoroids were additionally washed with PBS for 5 min three times and embedded in paraffin. Tumoroids sections (5 µm thickness) were deparaffinized and hydrated through xylenes and graded alcohol series. Antigen retrieval was performed by heating the specimens in Tris/EDTA buffer, pH 9.0 (Dako target retrieval solution S2367, DAKO, Carpenteria, CA, USA) using a microwave for 3 min for CD9 or by autoclaving in 0.01 M citrate buffer pH 6.0 (sodium citrate dihydrate, citric acid; Sigma Aldrich, USA) in a pressure cooker for 8 min for MMP3 and Ki-67. Sections were treated with blocking solution (Dako) for 30 min at RT, then incubated with primary antibodies; rabbit anti-CD9 (EPR2949, ab92726, Abcam, Cambridge, UK), rabbit anti-MMP3 (EP1186Y, ab52915, Abcam, Cambridge, UK), or rat anti-Ki-67 antibody (TEC-3, M7249, Dako); individually at 4 °C overnight. Then, sections were subsequently incubated with a secondary antibody goat anti-rabbit IgG, Alexa Fluor 488 (A-11034, Thermo Fisher, Waltham, MA, USA) for 1 h at RT. Then samples were counterstained with 1 mg/mL of DAPI (Dojindo Laboratories, Kumamoto, Japan). Fluorescent images were taken using a confocal laser scanning microscopy LSM780 (Carl Zeiss, Oberkochen, Germany) at Central Research Laboratory, Okayama University Medical School.

For IHC staining of Ki67, a biotinylated secondary antibody was applied for 30 min (Vector Lab, Burlingame, CA, USA) and the color was developed with 3, 3′-diaminobenzidine (DAB) (Histofine DAB substrate; Nichirei, Tokyo, Japan). Then, samples were counterstained with Myer’s hematoxylin and images were taken using an optical microscope BX53 (Olympus, Tokyo, Japan). To calculate the Ki-67 labeling index (%), we counted approximately 100 Ki67-positive cells in random five fields under the 40× objective. Areas with severe necrosis were avoided. The Ki-67 labeling index (%) was calculated by dividing the total Ki-67 positive cells by the total numbers of cells multiplied by 100.

The total tumoroid areas, as well as the area of necrotic regions were measured using Image J. The percentage of necrosis was calculated by dividing the total necrotic area by the total tumoroid area.

### 4.14. Hematoxylin and Eosin Staining

For tumoroid formation, LuM1 or MMP3-KO cells were cultured for 8 days on 6-well ULA plates at a density of 3.0 × 10^5^/3 mL mTeSR1/well. Then tumoroids were washed with PBS, fixed in 4% PFA for 10 min, and embedded in paraffin. Tumoroids sections (5 µm thickness) were deparaffinized in a series of xylene for 15 min, rehydrated in graded ethanol solutions, and washed well in distilled water. Then sections were incubated in Harris hematoxylin solution for 10 min and rinsed in tap water until the water was colorless. Finally, after sequential treatment with hydrogen chloride and 80% ethanol solution, sections were incubated in eosin for 7 min.

### 4.15. Tracing EV-Uptake In Vitro

Ten micrograms of tumoroid-derived EVs were incubated with 0.25 μM BODIPY TR Ceramide (Thermo Fisher, Waltham, MA, USA) for 20 min at 37 °C. Excessive BODIPY TR Ceramide was removed with Exosome Spin Columns (MW 3000) (Thermo Fisher, Waltham, MA, USA) [37]. Cells were seeded at a concentration of 5.0 × 10^3^ cells/200 μL mTeSR1 in a well of 96-well NCP. The next day, EVs were added at a final concentration of 5 μg/mL. The EVs-uptake was monitored over 24 h using the ArrayScan High Content Screening (HCS) system (Thermo Fisher, Waltham, MA, USA). The fluorescence intensity of each cell was determined using a filter set (485/594) for (GFP/ BODIPY TR). The average fluorescence intensity of the PBS treatment group at time point 0 h was evaluated as background and subtracted from raw values.

### 4.16. Statistical Analysis

Statistical significance was calculated using GraphPad Prism and Microsoft Excel. The difference between the sets of data was analyzed using ANOVA Tukey’s multiple comparisons test and all data are expressed as the mean ± standard deviation unless otherwise indicated.

## 5. Conclusions

Our study demonstrated that the loss-of-function of MMP3 significantly decreased the 3D-tumoroids formation in vitro, reduced tetraspanins (CD9 and CD63) in EVs, and resulted in destabilizing the EVs structural integrity. Moreover, we proved the successful labeling, exchanging of EVs between cells, and established a bidirectional EV transfer assay system. We confirmed the EVs-mediated molecular transfer of MMP3 into the MMP3-KO tumoroids under the 3D culture system. Also, we found that the addition of MMP3-enriched EVs (defined as oncosomes) fostered the tumorigenicity and increased the proliferation of MMP3-null cells. Thus MMP3-enriched oncosomes are highly transmissive and protumorigenic.

## Figures and Tables

**Table 1:** Comparison of particle size distributions between LuM1-EVs and MMP3-KO-EVs.

LuM1-EV				MMP3-KO-EV			
Peak	Diameter (nm)	Intensity (%)	Width (nm)	Peak	Diameter (nm)	Intensity (%)	Width (nm)
1	561.4	48.6	164.9	1	464.9	76.6	155.3
2	119.3	38.3	31.8	2	83.9	20.2	20.4
3	8.0	6.8	1.53	3	5,374	3.2	326.1

**Table 2:** Necrotic areas in the LuM1 tumoroid versus MMP3-KO tumoroid.

	LuM1	MMP3-KO
Total tumoroid area (μm^2^)	331,701	64,148
Number of necrotic areas	1	5
Sum of necrotic areas (μm^2^)	4807	9351
Necrosis %	1	15

## Supplementary Materials

The following are available online at https://www.mdpi.com/2072-6694/12/5/1260/s1. Figure S1. Full images of Western blotting of (A) MMP3, (B) CD9, (C) CD63, (D) β-actin, and (E) GAPDH, supporting Figure 1. Images from long and short exposure times are shown. The protein amount loaded from WCL and EV fractions was 10 µg per lane, while 5 µg per lane was loaded from the non-EV fractions. Figure S2. Images of (A) GAPDH Western blotting and (B) CBB staining of the SDS-PAGE. The amount of protein sample loaded for the CBB staining was 1µg per each lane. Figure S3. Column scatters plotting of the size of LuM1 tumoroids versus MMP3-KO tumoroids cultured for (A) 7 days or (B) 14 days in serum-containing or stemness media, supporting Figure 3. ** p < 0.01, **** p < 0.0001; ns, not significant. Figure S4. Evaluation of three different concentrations of LuM1-EVs on the MMP3-KO tumoroids growth. MMP3-KO tumoroids were treated with LuM1-EVs at a concentration of 1.25, 2.5, 5 µg/mL in the NCP-based 3D culture with the stemness-enhancing medium. Scale bar, 100 µm. (B,C) Tumoroid size quantification on (B) day 3 and (C) day 5 of the tumoroid formation periods. * p < 0.05, ** p < 0.01, *** p < 0.001; ns, not significant. Figure S5. Specificity of the MMP3 antibody. (A,B) MMP3 expression (green) in (A) positive (B) and negative controls; LuM1/palmT tumoroids were stained with or without the MMP3 antibody, then stained with the secondary AF488 antibody. Nuclei were stained blue with DAPI. Images were taken by CLSM. w/o Ab; without antibody Scale bar 100 µm.
