# Supplementary material for: Knockout of MMP3 Weakens Solid Tumor Organoids and Cancer Extracellular Vesicles
Source: Cancers (Basel). 2020 May 16;12(5):1260. doi: 10.3390/cancers12051260 (PMC7281240; doi:10.3390/cancers12051260)
Supplement: Supplementary file 1 [file cancers-12-01260-s001.zip › cancers-768555-supplementary.pptx]

## Slide 1
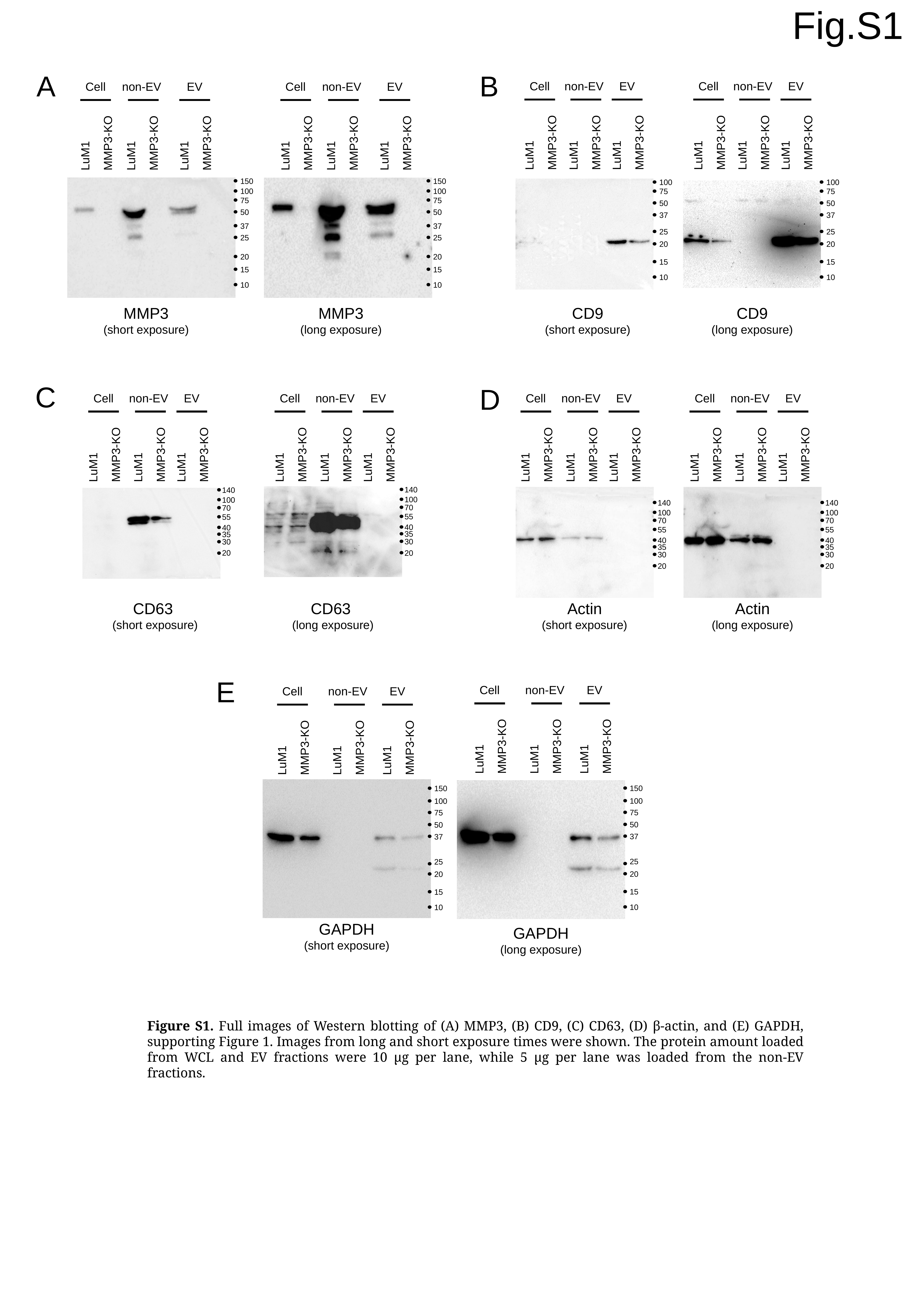

Fig.S1
A
B
Cell
non-EV
 EV
MMP3-KO
MMP3-KO
MMP3-KO
LuM1
LuM1
LuM1
Cell
non-EV
 EV
MMP3-KO
MMP3-KO
MMP3-KO
LuM1
LuM1
LuM1
Cell
non-EV
 EV
MMP3-KO
MMP3-KO
MMP3-KO
LuM1
LuM1
LuM1
Cell
non-EV
 EV
MMP3-KO
MMP3-KO
MMP3-KO
LuM1
LuM1
LuM1
150
100
75
50
37
25
20
15
10
150
100
75
50
37
25
20
15
10
100
75
50
37
25
20
15
10
100
75
50
37
25
20
15
10
MMP3
(short exposure)
MMP3
(long exposure)
CD9
(short exposure)
CD9
(long exposure)
C
D
Cell
non-EV
 EV
MMP3-KO
MMP3-KO
MMP3-KO
LuM1
LuM1
LuM1
Cell
non-EV
 EV
MMP3-KO
MMP3-KO
MMP3-KO
LuM1
LuM1
LuM1
Cell
non-EV
 EV
MMP3-KO
MMP3-KO
MMP3-KO
LuM1
LuM1
LuM1
Cell
non-EV
 EV
MMP3-KO
MMP3-KO
MMP3-KO
LuM1
LuM1
LuM1
140
100
70
55
40
35
30
20
140
100
140
100
70
55
40
35
30
20
140
100
70
55
40
35
30
20
70
55
40
35
30
20
CD63
(short exposure)
CD63
(long exposure)
Actin
(short exposure)
Actin
(long exposure)
E
Cell
non-EV
 EV
MMP3-KO
MMP3-KO
MMP3-KO
LuM1
LuM1
LuM1
Cell
non-EV
 EV
MMP3-KO
MMP3-KO
MMP3-KO
LuM1
LuM1
LuM1
150
100
75
50
37
25
20
15
10
GAPDH
(short exposure)
150
100
75
50
37
25
20
15
10
GAPDH
(long exposure)
Figure S1. Full images of Western blotting of (A) MMP3, (B) CD9, (C) CD63, (D) β-actin, and (E) GAPDH, supporting Figure 1. Images from long and short exposure times were shown. The protein amount loaded from WCL and EV fractions were 10 µg per lane, while 5 µg per lane was loaded from the non-EV fractions.

## Slide 2
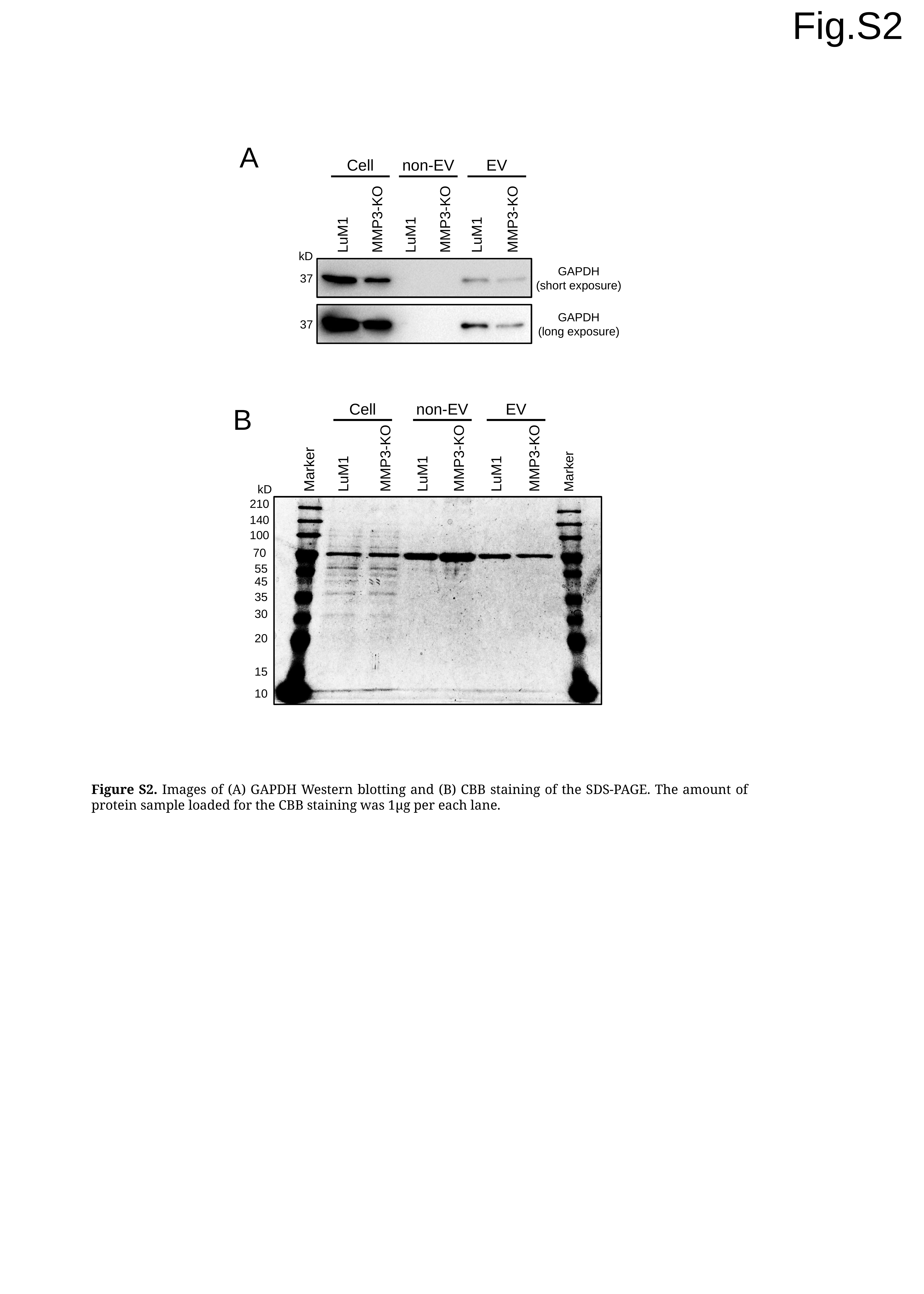

Fig.S2
A
Cell
non-EV
 EV
MMP3-KO
MMP3-KO
MMP3-KO
LuM1
LuM1
LuM1
kD
GAPDH
(short exposure)
37
GAPDH
(long exposure)
37
Cell
non-EV
 EV
B
MMP3-KO
MMP3-KO
MMP3-KO
Marker
LuM1
LuM1
LuM1
Marker
kD
210
140
100
70
55
45
35
30
20
15
10
Figure S2. Images of (A) GAPDH Western blotting and (B) CBB staining of the SDS-PAGE. The amount of protein sample loaded for the CBB staining was 1µg per each lane.

## Slide 3
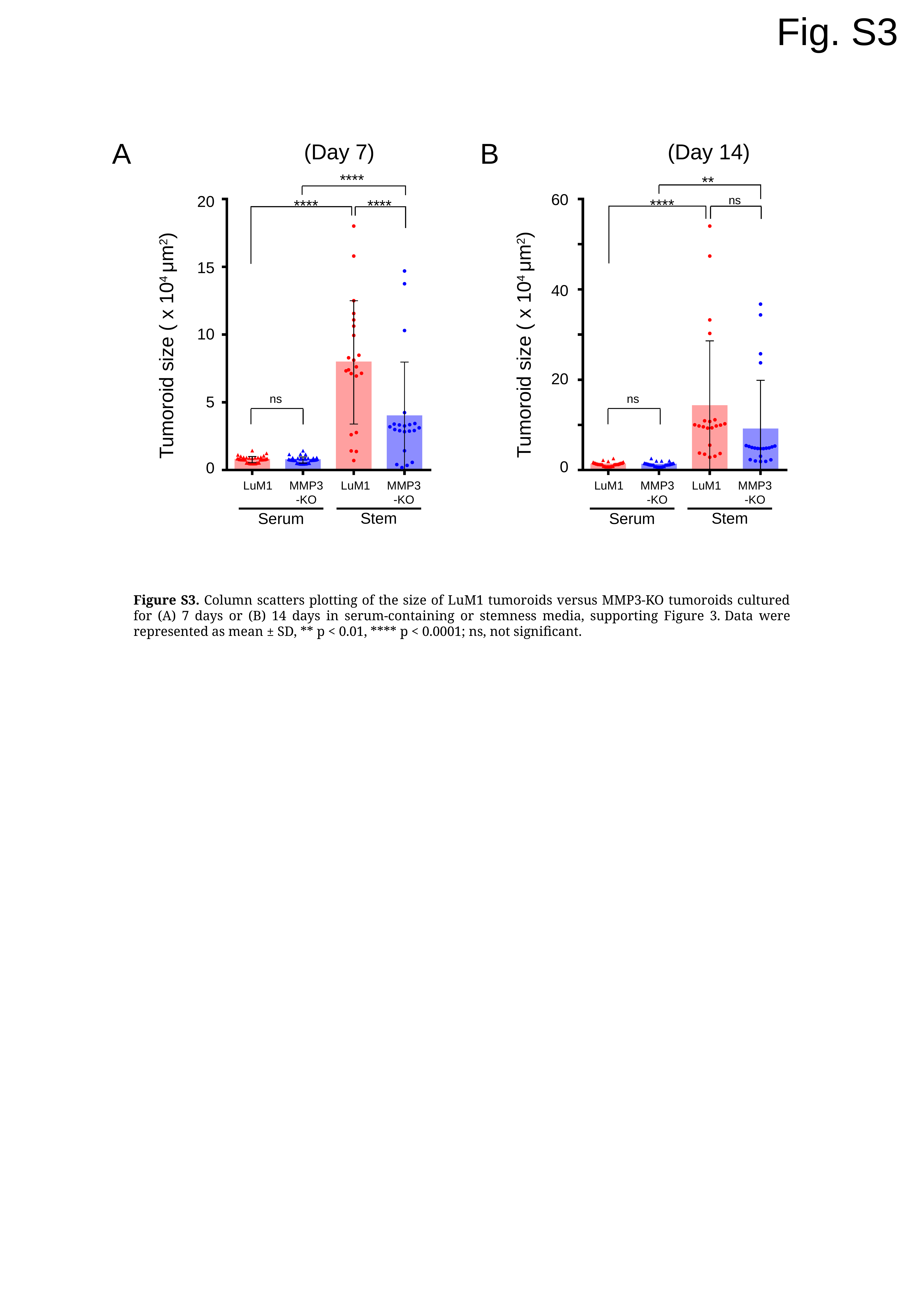

Fig. S3
A
B
(Day 7)
(Day 14)
****
**
60
40
20
0
20
15
10
5
0
ns
****
****
****
Tumoroid size ( x 104 μm2)
Tumoroid size ( x 104 μm2)
ns
ns
LuM1
MMP3
-KO
LuM1
MMP3
-KO
Stem
Serum
LuM1
MMP3
-KO
LuM1
MMP3
-KO
Stem
Serum
Figure S3. Column scatters plotting of the size of LuM1 tumoroids versus MMP3-KO tumoroids cultured for (A) 7 days or (B) 14 days in serum-containing or stemness media, supporting Figure 3. Data were represented as mean ± SD, ** p < 0.01, **** p < 0.0001; ns, not significant.

## Slide 4
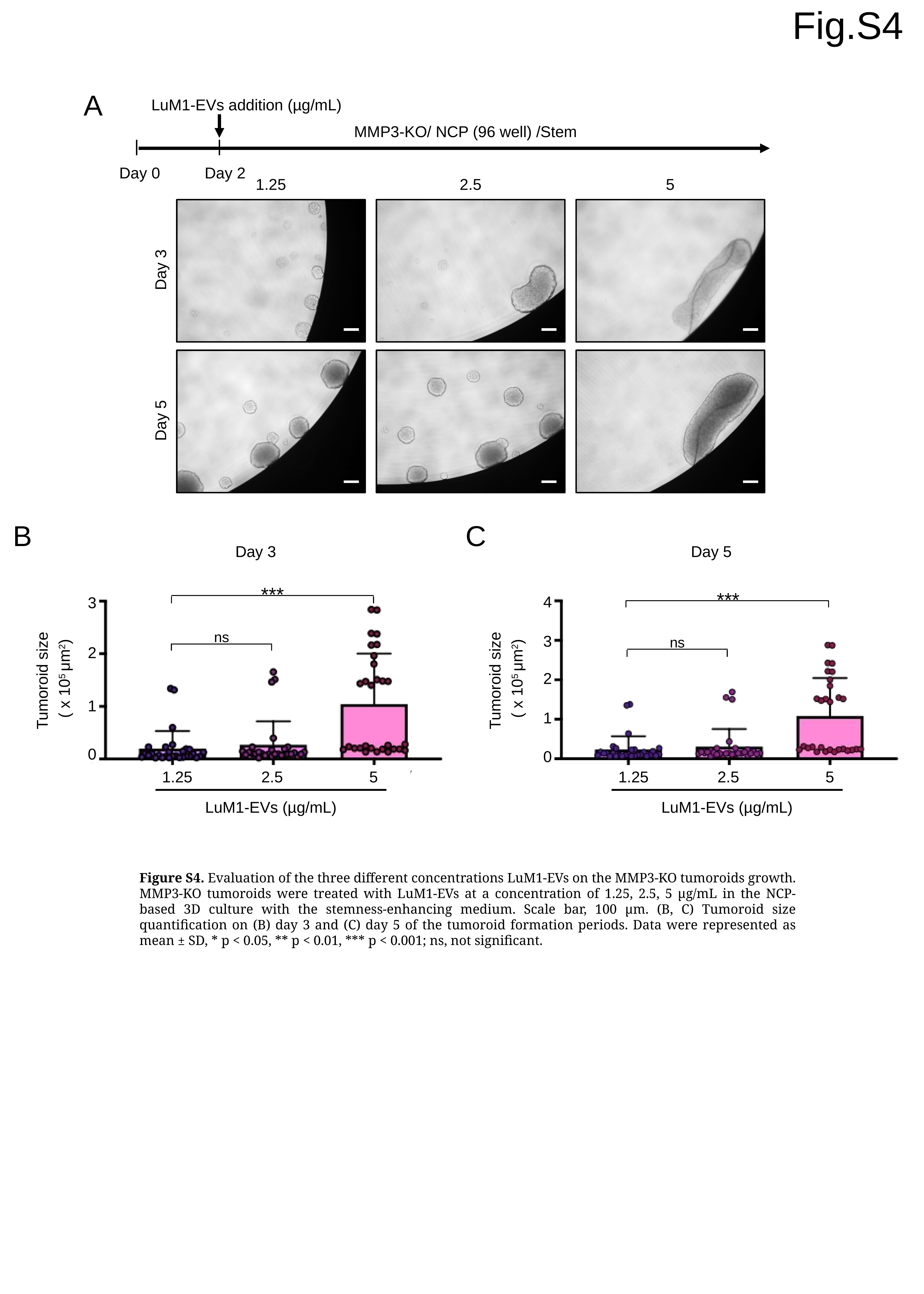

Fig.S4
A
LuM1-EVs addition (µg/mL)
MMP3-KO/ NCP (96 well) /Stem
Day 0
Day 2
1.25
2.5
5
Day 3
Day 5
B
C
Day 3
Day 5
***
***
4
3
2
1
0
3
2
1
0
Tumoroid size
( x 105 μm2)
Tumoroid size
( x 105 μm2)
Don’t you cut off small tumoroids?
No, I measured 1 field/conc, I remeasured again (3 fields/conc.). I do not have pictures for other days, to make it like S1 &3, It would be better if we remove it.
ns
ns
1.25
2.5
5
LuM1-EVs (µg/mL)
1.25
2.5
5
LuM1-EVs (µg/mL)
Figure S4. Evaluation of the three different concentrations LuM1-EVs on the MMP3-KO tumoroids growth. MMP3-KO tumoroids were treated with LuM1-EVs at a concentration of 1.25, 2.5, 5 µg/mL in the NCP-based 3D culture with the stemness-enhancing medium. Scale bar, 100 µm. (B, C) Tumoroid size quantification on (B) day 3 and (C) day 5 of the tumoroid formation periods. Data were represented as mean ± SD, * p < 0.05, ** p < 0.01, *** p < 0.001; ns, not significant.

## Slide 5
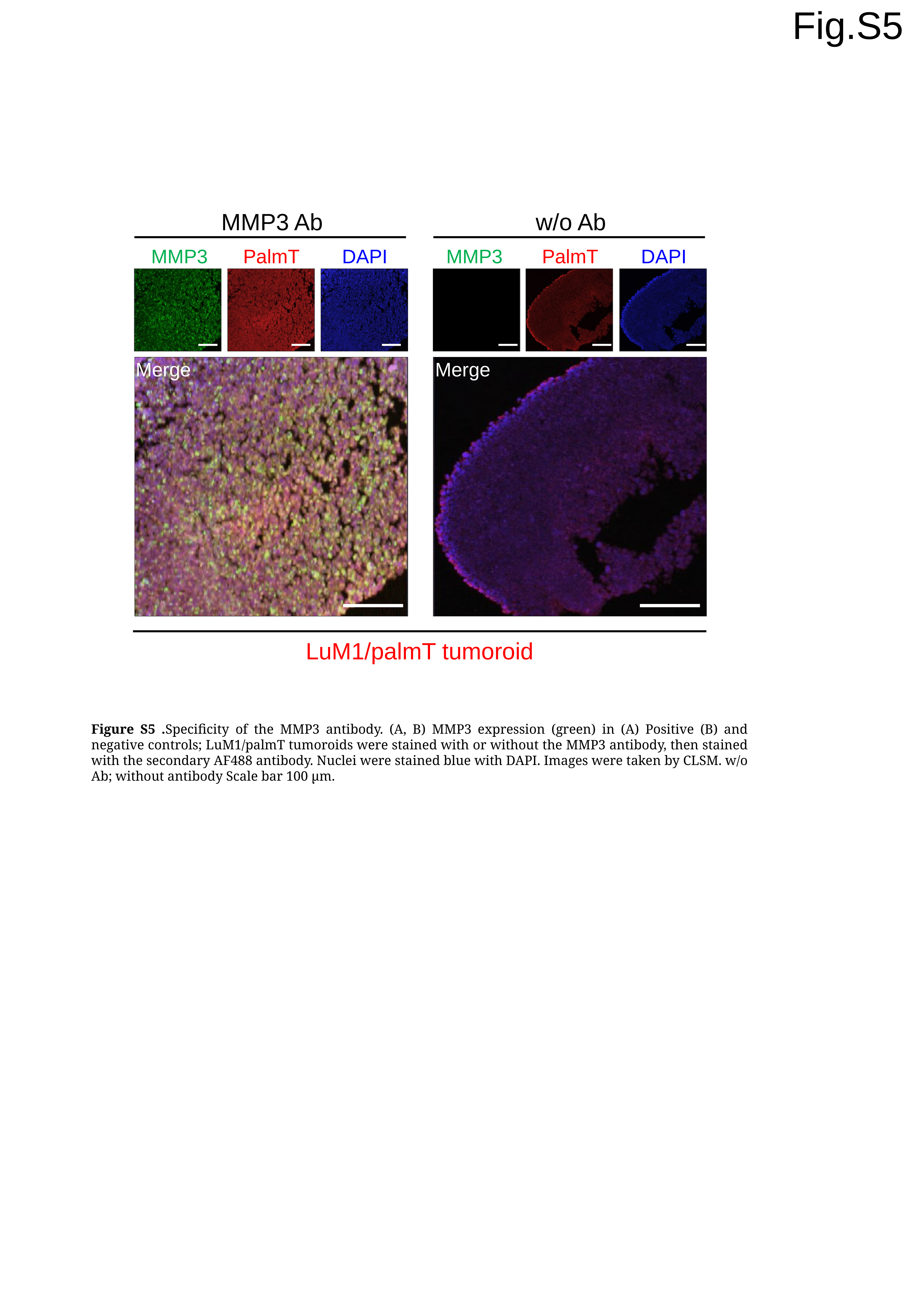

Fig.S5
MMP3 Ab
w/o Ab
MMP3
PalmT
DAPI
MMP3
PalmT
DAPI
Merge
Merge
LuM1/palmT tumoroid
Figure S5 .Specificity of the MMP3 antibody. (A, B) MMP3 expression (green) in (A) Positive (B) and negative controls; LuM1/palmT tumoroids were stained with or without the MMP3 antibody, then stained with the secondary AF488 antibody. Nuclei were stained blue with DAPI. Images were taken by CLSM. w/o Ab; without antibody Scale bar 100 µm.
